# Multi-omics approaches explain the growth-promoting effect of the apocarotenoid growth regulator zaxinone in rice

**DOI:** 10.1038/s42003-021-02740-8

**Published:** 2021-10-25

**Authors:** Jian You Wang, Saleh Alseekh, Tingting Xiao, Abdugaffor Ablazov, Leonardo Perez de Souza, Valentina Fiorilli, Marita Anggarani, Pei-Yu Lin, Cristina Votta, Mara Novero, Muhammad Jamil, Luisa Lanfranco, Yue-Ie C. Hsing, Ikram Blilou, Alisdair R. Fernie, Salim Al-Babili

**Affiliations:** 1 The BioActives Lab, Center for Desert Agriculture (CDA), Biological and Environment Science and Engineering (BESE), King Abdullah University of Science and Technology, Thuwal, 23955-6900 Saudi Arabia; 2 Max-Planck-Institute of Molecular Plant Physiology, Am Mühlenberg 1, 14476 Potsdam-Golm, Germany; 3 Center of Plant Systems Biology and Biotechnology, 4000 Plovdiv, Bulgaria; 4 The Laboratory of Plant Cell and Developmental Biology (LPCDB), Biological and Environment Science and Engineering (BESE), King Abdullah University of Science and Technology, Thuwal, 23955-6900 Saudi Arabia; 5 Department of Life Sciences and Systems Biology, University of Turin, Turin, Italy; 6 Institute of Plant and Microbial Biology, Academia Sinica, No. 128, Section 2, Yien-Chu-Yuan Road, Taipei, 11529 Taiwan

**Keywords:** Plant hormones, Plant development, Plant physiology

## Abstract

The apocarotenoid zaxinone promotes growth and suppresses strigolactone biosynthesis in rice. To shed light on the mechanisms underlying its growth-promoting effect, we employed a combined omics approach integrating transcriptomics and metabolomics analysis of rice seedlings treated with zaxinone, and determined the resulting changes at the cellular and hormonal levels. Metabolites as well as transcripts analysis demonstrate that zaxinone application increased sugar content and triggered glycolysis, the tricarboxylic acid cycle and other sugar-related metabolic processes in rice roots. In addition, zaxinone treatment led to an increased root starch content and induced glycosylation of cytokinins. The transcriptomic, metabolic and hormonal changes were accompanied by striking alterations of roots at cellular level, which showed an increase in apex length, diameter, and the number of cells and cortex cell layers. Remarkably, zaxinone did not affect the metabolism of roots in a strigolactone deficient mutant, suggesting an essential role of strigolactone in the zaxinone growth-promoting activity. Taken together, our results unravel zaxinone as a global regulator of the transcriptome and metabolome, as well as of hormonal and cellular composition of rice roots. Moreover, they suggest that zaxinone promotes rice growth most likely by increasing sugar uptake and metabolism, and reinforce the potential of this compound in increasing rice performance.

## Abstract (web summary)

Wang et al. report zaxinone as a global regulator of the transcriptome and metabolome, as well as of hormonal and cellular composition of rice roots. This study shows that zaxinone promotes rice growth by enhancing root sugar uptake and metabolism and modulation of cytokinin content, indicating the potential application of this compound in increasing rice performance.

## Introduction

Carotenoids are widespread pigments fulfilling vital functions in plants, by protecting the photosynthetic apparatus from photo-oxidation and harnessing light energy^1^. In addition, they are the precursor of a structurally diverse set of metabolites, generally called apocarotenoids, which include volatiles, colorants, signaling/regulatory molecules, and hormones. Apocarotenoids arise through oxidative break down of carotenoids, which is initiated by reactive oxygen species (ROS) attack or catalyzed by Carotenoid Cleavage Dioxygenases (CCDs), an evolutionarily conserved family of non-heme Fe^2+^-dependent enzymes^2–4^. The primary cleavage products are frequently modified by different enzymes, before acquiring their biological function. For instance, the apocarotenoid plant hormone strigolactone (SL) is formed by the sequential action of CCD7, CCD8, more axillary growth1 (MAX1, a cytochrome P450 monooxygenase), and other enzymes^4–8^. SLs and abscisic acid (ABA), a further carotenoid-derived plant hormone, are key metabolites in establishing plant’s response to abiotic and biotic stress, and major determinants of plant development^4,9^. Besides, SLs modulate plant’s architecture in response to nutrients availability, particularly phosphorus, and mediate, when released in the rhizosphere, the communication with arbuscular mycorrhizal fungi that supply plants with water and minerals^10,11^. However, SLs are also perceived by seeds of parasitic plants, such as *Striga*, which use them as germination stimulus ensuring the availability of a host required for the survival of these obligate parasitic weeds^5^. To fulfill their role in plant growth and development, SLs are embedded in a complex hormonal network in which they affect and are influenced by the activity of other plant hormones. Indeed, auxin and gibberellins were reported to interact with SL biosynthesis or signaling in rice and *Arabidopsis*^12^. Vice versa, it was shown that SLs enhance cytokinins (CKs) catabolism by modulating *CYTOKININ OXIDASE/DEHYDROGENASE 9* (*CKX9*) expression to inhibit rice tillering^13^. Similarly, CKs and SLs exert opposite effects on rice mesocotyl elongation^14,15^.

Metabolism is a central process required for the uptake and utilization of energy and nutrients to ensure the survival, reproduction, growth, and development of living organisms^16^. Thus, primary metabolites such as sugars, amino acids, nucleotides, organic acids, and fatty acids are essential for maintaining cellular homeostasis and for organismal life^17^. In fact, metabolites are direct physiological signatures that are highly correlated with end-phenomes in plants^17,18^. In addition, some primary metabolites act as signaling molecules regulating plant growth and development. For instance sugars, such as sucrose, interact with different plant hormones and regulate bud development and shoot branching by modulating the signaling of auxin and SLs^12,19,20^.

Besides the established plant hormones ABA and SLs, the apocarotenoid family includes growth regulators, such as anchorene that specifically promotes the growth of anchor roots in *Arabidopsis*^21^, and signaling molecules, such as cyclocitral that mediates the response of plants to high-light and drought stress, and regulates roots growth^4,22^. Recently, we have identified zaxinone, an apocarotenoid hormone candidate, as a metabolite required for proper rice growth and development, and characterized a rice CCD, called ZAXINONE SYNTHASE (ZAS), involved in its biosynthesis^23^. A rice loss-of-function *zas* mutant showed shoot and root growth impairment, a lower root zaxinone level, and higher SL content in roots and root exudates. Exogenous application of zaxinone rescued several *zas* phenotypes and resulted in a decrease in SL content and release, and in promoted root growth^23,24^. Treatment of WT seedlings with zaxinone led also to an obvious increase in root growth and a suppression of SL formation^23,24^. Transcript analysis showed that zaxinone is a negative regulator of SL biosynthesis at the transcript level. However, zaxinone application did not enhance root growth in SL biosynthesis and perception rice mutants, which indicates an interaction between zaxinone and SLs and suggests the requirement of a functional SL pathway for zaxinone’s growth-promoting activity^23^.

In the current study, we set out to understand how zaxinone promotes rice growth. For this purpose, we characterized its effect on rice primary metabolism and transcriptome in WT and the SL-deficient *d17* mutant rice plants, and determined zaxinone’s impact on hormone content and root anatomy. Our results unraveled enhanced root sugar metabolism as a major reason for zaxinone growth-promoting activity and point to modulation of cytokinin content as a likely reason for increased root cell division activity and enhanced number of cortex cell layers, which we observed in roots.

## Results

### Zaxinone treatment increases sugar content and metabolism

**Fig. 1:**
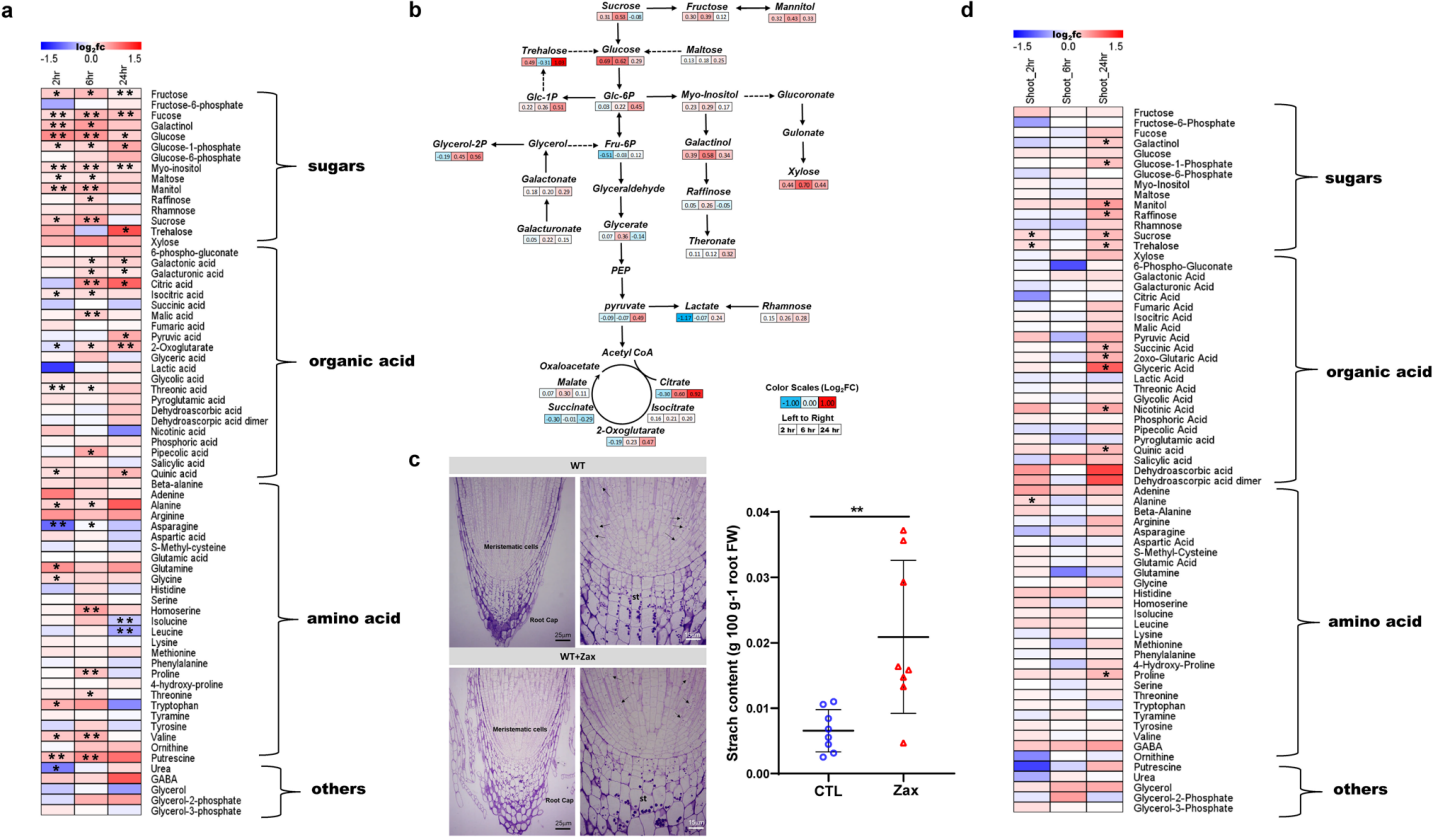
The dynamic primary metabolite profiles upon zaxinone treatment. Primary metabolites extracted from roots for GC–MS, which were annotated and listed in Supplementary Data 6. Three independent harvests were concurrently subjected to GC–MS analysis. *n* ≥ 4 biological replicates. **a** Heatmap of root tissue showing relative accumulation of each metabolite as compared to those in control plants. For each metabolite, the value of the corresponding wild type was set to 1. Asterisks indicate statistically significant differences as compared to control by *t*-test (*p < 0.05, **p < 0.01). **b** The scheme of major metabolic changes in the sugar-related metabolites after zaxinone treatment, which adapted from **a**. Blue and red color depict a decrease and increase in metabolic levels compared to the non-treated root samples, respectively. The data are presented as log2 (fold changes) from left to right as follows: 2, 6, and 24 h. **c** Longitudinal-sections of WT and WT treated with 5 µM zaxinone roots tips after resin-embedding and staining by the Periodic Acid-Schiff (PAS) reaction for the visualization of amyloplasts. At higher magnification (15 µm) the statoliths (st) in the root cap, as well as some tiny amyloplasts (arrows), are present in the cytoplasm of meristematic cells. The starch level was quantified in the root tissues. Bar presented as mean ± SD, *n* = 8 biological replicates. **d** Heatmap of shoot tissue of relative accumulated metabolites in comparison with control. For each metabolite, the value of the corresponding wild type was set to 1. Asterisks indicate statistically significant differences as compared to wild type by *t*-test (*p < 0.05, **p < 0.01). CTL control, Zax zaxinone.

To determine the effects of zaxinone on rice at a metabolomic and transcriptomic level and to get an insight into the dynamics of triggered changes, we grew rice seedlings hydroponically, applied the compound at a 5 µM concentration into the growth medium, and collected root and shoot samples 2, 6 and 24 h after application. A scheme of the experimental design is shown in Supplementary Fig. 1. Gas chromatography–mass spectrometry (GC–MS) analysis of primary metabolites in treated roots revealed an up to a 1.5-fold increase in the level of many sugars, glycolysis- and tricarboxylic acid (TCA)-cycle intermediates, such as glucose, citric acid, and 2-oxoglutarate (Fig. 1a). The position of these metabolites in cellular sugar catabolism pathways is shown in Fig. 1b. At the same time point, i.e., 6 h after zaxinone application, we also observed an increase in the content of most of the free amino acids and many of other analyzed primary metabolites. However, the content of some amino acids, e.g., leucine, dropped at the 24 h time point to below control level (Fig. 1a). Principal component analysis (PCA) revealed that the zaxinone effect on root metabolome was more pronounced at 6 and 24 h, compared with the early 2 h time point, with a peak of primary metabolites accumulation at 6 h (Fig. 1a and Supplementary Figs. 2a, Supplementary Fig. 3). In more detail, we observed a significant increase in the levels of the major sugars sucrose, glucose, and fructose at 2 and 6 h after application of zaxinone, which was followed by a sharp decrease at 24 h. In contrast, trehalose showed the highest increase after 24 h, similar to maltose, glucose-1-phosphate, and glucose-6-phosphate, which indicates a biphasic response to zaxinone application. In shoot tissues, we also detected a quick enhancement in the level of sucrose and trehalose 2 h after zaxinone application. However, many other primary metabolites, including several sugars, TCA intermediates, and free amino acids, showed an increase only at the late, 24 h, time point (Fig. 1d and Supplementary Figs. 2b,  4). These results suggest that zaxinone application to roots in hydroponically grown seedlings causes a rapid global change in the primary metabolism of roots and, with a slight delay, in shoots. In particular, it affects sugar content and catabolism, which are essential for adenosine triphosphate (ATP) generation and supply of C skeletons for cellular building blocks.

Assuming that excess sugars may be stored as starch, we measured the starch level in roots of hydroponically grown seedlings after two weeks of treatment with zaxinone (5 µM). Indeed, we detected around two-fold higher starch content in treated roots (Fig. 1c), compared to the mock condition. Taking into consideration that plants produce sugars through photosynthesis, we investigated the effect of zaxinone on this process. For this purpose, we performed a time-course measurement of chlorophyll content and stomatal conductance, two parameters of photosynthetic activity, in a 3-weeks-old hydroponic grown rice plant treated with 5 µM zaxinone under greenhouse condition. As shown in Supplementary Fig. 5a, b, we observed an enhancement of both parameters in leaves of treated rice plants, which indicated an increased photosynthetic activity and may explain the elevated sugar levels.

### Zaxinone application increases transcript level of genes involved in root sugar metabolism

**Fig. 2:**
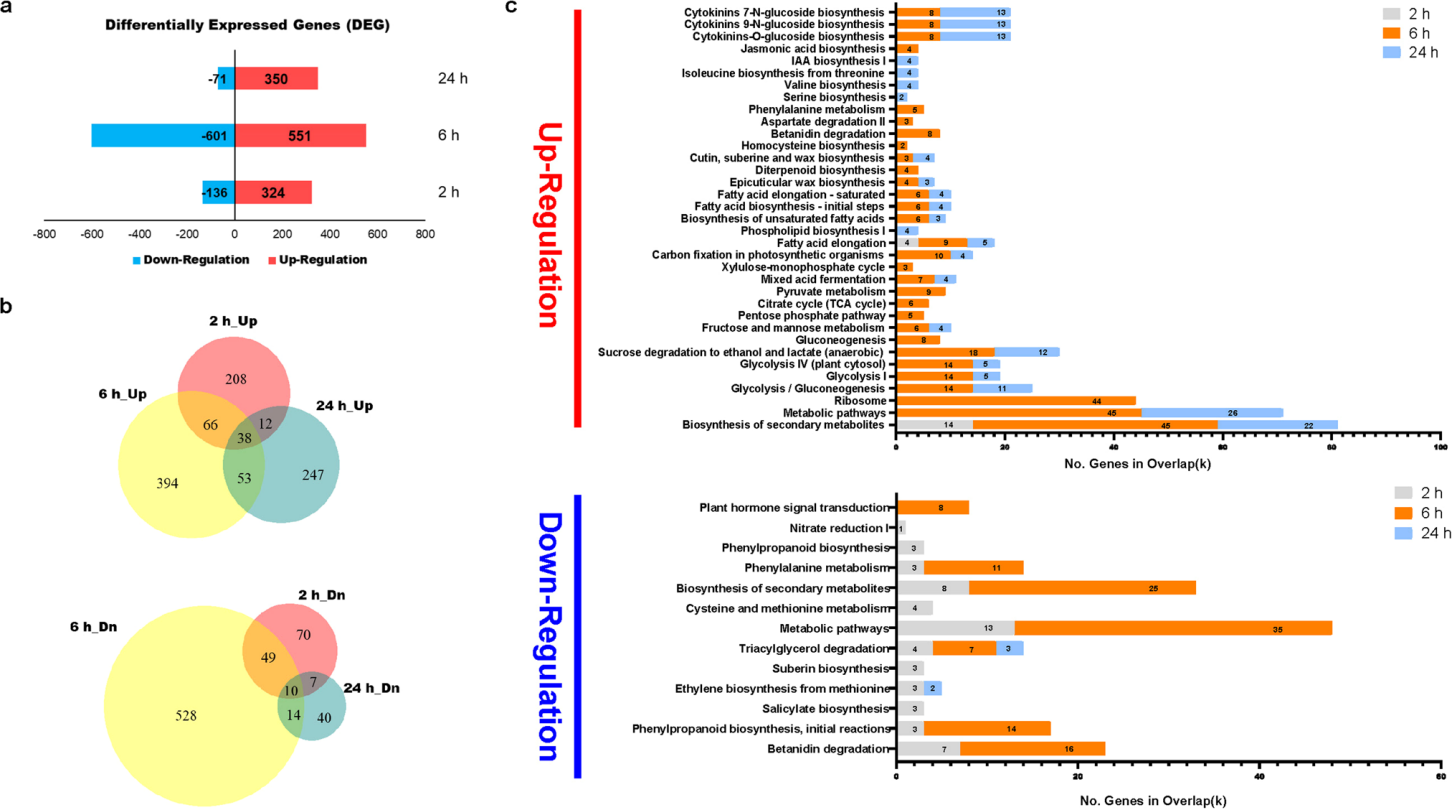
Analysis of differentially expressed genes (DEGs) in response to zaxinone at different time points. **a** Numbers of the significantly expressed genes upon zaxinone treatment (FDR < 0.05). The numbers on the vertical axis represent the three time points while the horizontal axis reflects the numbers of up- and down-regulated genes. Up- and down-regulated genes are shown in red and blue bars, respectively. **b** Venn diagrams showing the numbers of down (Dn) and upregulated (Up) genes that overlap between different time points. **c** Kyoto Encyclopedia of Genes and Genomes (KEGG) and PlantCyc pathway enrichment analysis for up- and down-regulated genes, which were analyzed by The Plant GeneSet Enrichment Analysis Toolkit (PlantGSEA) (http://structuralbiology.cau.edu.cn/PlantGSEA/index.php).

We also analyzed the impact of zaxinone treatment on rice transcriptome, using RNA sequencing (RNA-Seq). A heatmap visualization of mean-centered, normalized log-expression values for correlated highly variable genes (HVGs) of the RNA-Seq data confirmed the high quality of each replicate, which was supported by the PCA plots of HVGs (Supplementary Fig. 6). A Volcano plot of differentially expressed genes (DEGs), following Deseq2 analysis, revealed the gene expression pattern at different cutting points with log_2_FoldChange, adjusted *Q*-value (False discovery rate, FDR), or a combination of both (Supplementary Fig. 7). In order to have a better picture of the transcriptome, we decided to use the FDR < 0.05 as a criterion for further analysis (Supplementary Data 1). Zaxinone application led to significant changes in the transcriptome over time, by increasing the transcript level of 324, 551, and 350 genes after 2, 6, and 24 h, respectively, including 38 genes that showed an induction at the three time points. The application also decreased the transcript level of 136 (2 h), 501 (6 h), and 71 (24 h) genes, ten of which were downregulated at the three time points (Fig. 2a, b). To validate the RNA-Seq data, we determined the transcript level of 15 selected genes that showed low to high response to zaxinone treatment, by qRT-PCR. The resulting correlation analysis (*R*^2^ = 0.87–0.93) indicated that the RNA-Seq dataset was highly reliable and thus appropriate for pathway enrichment analysis (Supplementary Fig. 8). Gene Ontology (GO term) analysis of molecular function and biological process showed that most of the genes regulated by zaxinone were related to metabolic processes [upregulation: 45 (2 h), 108 (6 h), and 50 (24 h) genes; downregulation: 16 (2 h), 70 (6 h), and 8 (24 h) genes] and catalytic activity [upregulation: 72 (2 h), 148 (6 h), and 90 (24 h) genes; downregulation: 36 (2 h), 124 (6 h), and 22 (24 h) genes] (Supplementary Data 2). Further enrichments with Kyoto Encyclopedia of Genes and Genomes (KEGG) and PlantCyc pathway unraveled the induction of genes mediating several annotated sugar metabolism pathways, including pyruvate metabolism, citric acid cycle, sucrose degradation, glycolysis, and gluconeogenesis, particularly 6 h after zaxinone application (Fig. 2c), which is in line with the change in the profile of primary metabolites (Fig. 1a). We also confirmed the annotated pathways (the plant glycolytic pathway and the TCA cycle) by MapMan software (Supplementary Fig. 9). To validate these changes, we chose ten genes from the OyzaCyc 6.0 database, which are involved in root glycolysis (Supplementary Data 3), and validated their expression pattern by performing qRT-PCR analysis of the same samples used for the RNA-seq experiment (Supplementary Fig. 10). Results obtained correlated with and explained the increase in sugar metabolites in root tissues. In contrast to roots, we detected a less significant impact on the shoot transcriptome presented in the PCA plots and DEGs analysis (Supplementary Fig. 11 and Supplementary Data 4), which may be anticipated given that the effect of zaxinone was mainly visible in root tissues, when using the hydroponic system. To further confirm the results, we performed a parallel transcript analysis with roots of WT and the *zas* mutant that contains less zaxinone and displays retarded growth (Supplementary Fig. 12a). We observed an upregulated transcript pattern of five glycolytic genes following the zaxinone treatment (Supplementary Fig. 12b), which could explain the capability of zaxinone in rescuing *zas* phenotype. Taken together, the transcriptome analysis supported the hypothesis that the growth-promoting effect is strongly linked with an increase of sugar metabolism in rice roots.

### Zaxinone application does not induce sugar metabolism in the absence of strigolactones

**Fig. 3:**
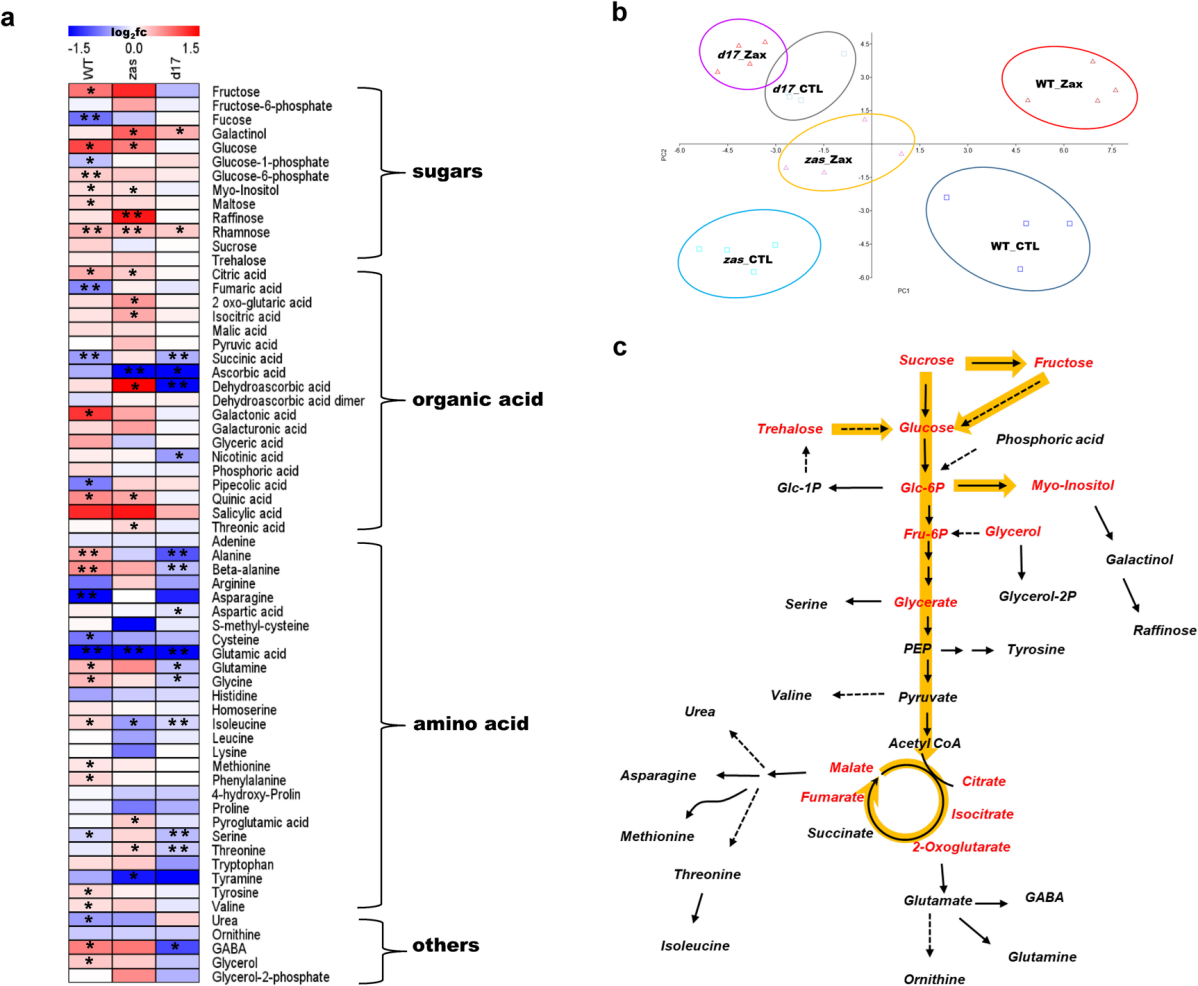
The profile of primary metabolites in roots of WT, *zas*, and *d17* mutant. Primary metabolites extracted from roots for GC-MS, which were annotated and listed in Supplementary Data 7. *n* = 4 biological replicates. **a** Heatmap of root tissue showing relative accumulation of each metabolite as compared to those in control plants. For each metabolite, the value of the corresponding control was set to 1. Asterisks indicate statistically significant differences as compared to control by *t*-test (*p < 0.05, **p < 0.01). **b** Principal component analysis (PCA) of root metabolites was performed using Past3 software. **c** The scheme of major metabolic changes in central metabolism after zaxinone treatment, which adapted from a. Orange arrows indicate sugar-related metabolites that mainly accumulated upon zaxinone treatment in WT and/or the *zas* mutant, but not in *d17*. CTL control, Zax zaxinone.

In our previous study, we showed that zaxinone application did not promote root growth in rice SL biosynthesis and perception mutants, indicating that zaxinone’s growth-promoting effect depends on functional SL biosynthesis and perception^23^. This opened the question of whether the changes in sugar metabolism caused by zaxinone are also linked to SLs. To answer this question, we applied zaxinone to *d17* and *zas* mutants and the corresponding WT varieties for 6 h, following the experimental design shown in Supplementary Fig 1, and analyzed the metabolome of collected root tissues. Results of metabolome analysis confirmed the accumulation of sugars and TCA cycle metabolites upon zaxinone treatment in both WT and *zas* mutants, while this response was largely absent in the *d17* mutant (Fig. 3), demonstrating that the sensitivity of sugar metabolism towards zaxinone application depends on the presence of a functional SL biosynthetic pathway.

Lipids are further important metabolites required for plant’s growth. To assess the effect of zaxinone on lipid metabolism, we analyzed the lipid profile of treated root samples 6 h after zaxinone application. However, we did not detect a positive effect of zaxinone on lipid metabolism (Supplementary Fig. 13).

### Zaxinone application promotes cell division in the root apical meristem and increases the number of cortex layers

**Fig. 4:**
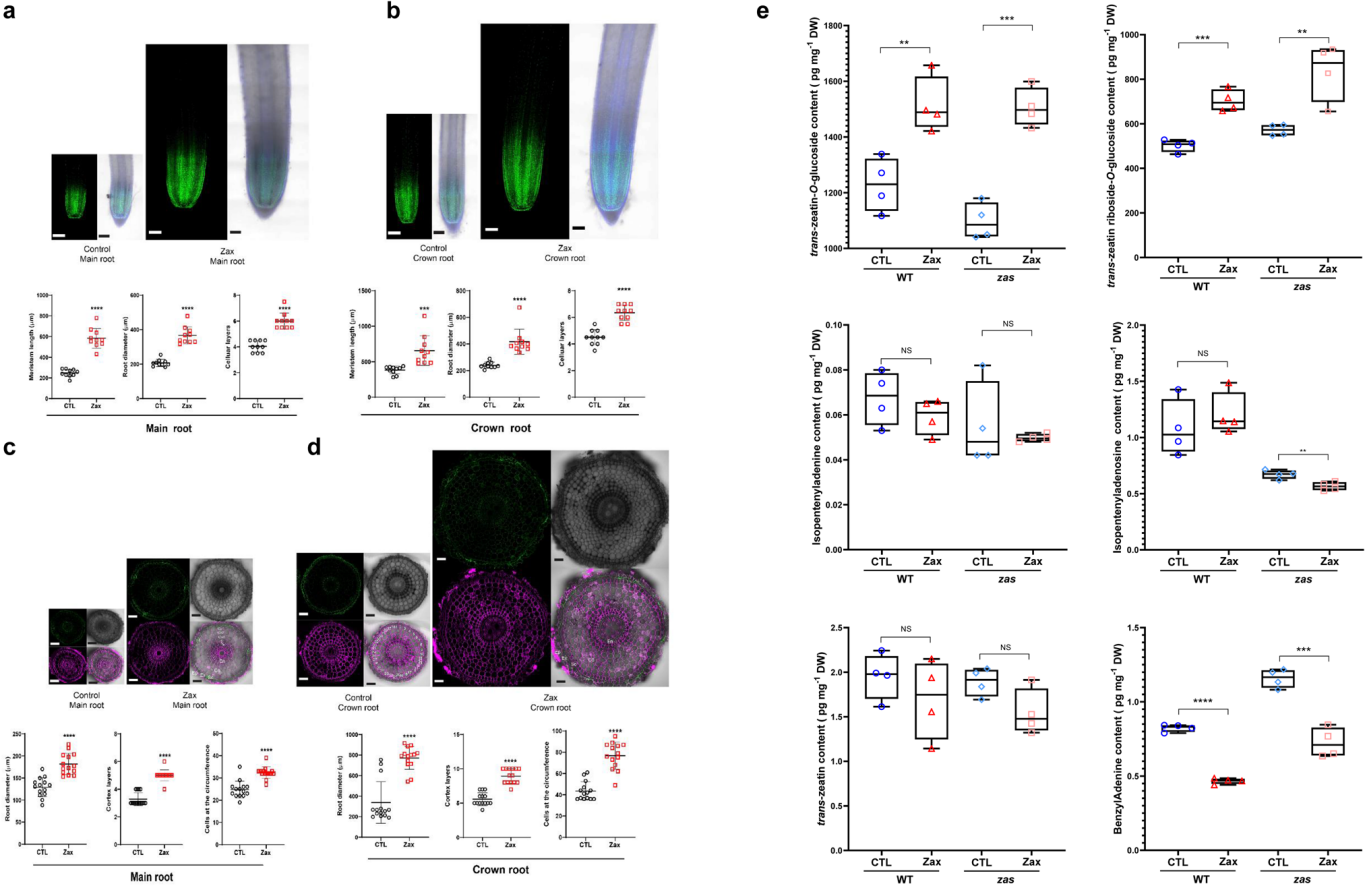
Characterization of root development at cellular level upon zaxinone treatment. **a**, **b** Ethynyl deoxyuridine (EdU) staining for cell proliferation analysis. Confocal images of rice root showing dividing cells as captured by EdU staining in Zeiss LSM 710 inverted confocal microscope. Root meristem length, root diameter, and cell layers (counted from epidermis to vascular tissue) in both primary roots and crown roots after 5 µM zaxinone treatment (twice per week) in Nipponbare WT rice seedlings. Dividing EdU-stained nuclei are shown in green; nuclei counterstained with Hoechst 33258 are shown in magenta. Images were acquired using the tile scan function in the Zen software with automatized stitching. Regions of interest were divided into multiple tiles and imaged individually. The tiles were then combined via automatic stitching to create a large overview image. Images are representative of the total number (*n* = 10) of seedlings that were studied. Scale bar: 50 µm. **c**, **d** Cross section of the mock and zaxinone-treated roots stained with SCRI Renaissance 2200. Magenta indicates the cell wall staining; green shows the auto-fluorescence marking lignin and suberin deposition (*n* = 14 biological replicates). Examples of cell layer and circumference cell number count are indicated in the cross section d, scale bar: 50 µm. **e** Quantification of cytokinins in root tissues of WT and *zas* mutant after 2-week zaxinone treatment. Box plot presented as min to max, *n* = 4 biological replicates. Asterisks indicate statistically significant differences as compared to control by *t*-test (*p < 0.05, **p < 0.01; ***p < 0.001; ****p < 0.0001). CTL control, Zax zaxinone. Ep epidermis, Ex exodermis, Sc sclerenchyma, Co cortex, En endodermis.

It can be assumed that the growth-promoting effect of zaxinone in rice roots is caused by an increase in cell number and/or size. To determine changes at a cellular level, we applied zaxinone (at 5 µM concentration) to hydroponically grown rice seedlings for 2 weeks and investigated the roots using cotton blue staining. As shown in Supplementary Fig. 14, the treatment with zaxinone enhanced the length of the root apex, suggesting an increase of cell division or cell elongation activity. To test this hypothesis, we used 5-Ethynyl-2′-deoxyuridine (EdU) staining that visualizes proliferating cells and can be monitored by a fluorescent dye. This experiment revealed that the meristem length and diameter, as well as the number of cell layers (counted from epidermis to vascular tissue), increased upon zaxinone application in both primary and the longest crown roots (Fig. 4a, b). To confirm the increase in the number of cell layers, we performed cross sections of primary and the longest crown roots of treated WT and *zas* mutant seedlings, by staining the cell wall with SCRI Renaissance 2200. In the main root cortex as well as in the longest crown roots, zaxinone application led to a remarkable increase in the number of cortex layers from around three to around five, and of the number of cells in the circumference by ten, which caused an around 50 µm enlargement of root diameters (Fig. 4c, d). Moreover, the enhancement in the number of cortex layers, root diameters, and cell numbers upon zaxinone application was much more conspicuous in the crown roots of *zas* mutant (Supplementary Fig. 15).

### Zaxinone enhances cytokinin glycosylation in rice roots

The changes in root morphology at the cellular level indicate that zaxinone may affect the hormonal composition in roots, in addition to its role in determining SL biosynthesis and sugar metabolism. Analysis of the RNA-Seq data indicated that zaxinone might affect several genes related to hormone metabolism, including genes involved in jasmonic acid and auxin biosynthesis and in the glycosylation of CKs (Fig. 2c). Therefore, we determined the changes in the hormone profile and content of abscisic acid (ABA), gibberellin (GA), auxin (IAA), salicylic acid (SA), jasmonic acid (JA), and cytokinins (CKs: *trans*-zeatin, isopentyladenine, isopentyladenosine, and benzyladenine) in rice roots 2, 6, and 24 h after zaxinone application. We did not detect significant changes in the levels of GA, IAA, or SA, compared to the control, but observed an increase in ABA and JA levels at 2 and 6 h, respectively (Supplementary Fig. 16). Notably, the application of zaxinone led to a significant decrease in the content of isopentenyladenosine in all treated samples, a reduction of isopentenyladenine and benyzladenine in the 6, and 6 and 24 h samples, respectively, and an increase in levels of glycosylated, inactive *trans*-zeatin forms. We also observed an increase in *trans*-zeatin content 2 h after application (Supplementary Fig. 17). These data indicate that zaxinone may regulate the abundance and pattern of CKs. To gain insights into the long-term effect of zaxinone on CKs level, we applied the compound for two weeks and quantified the hormone. In this experiment, we also included the *zas* mutant that showed at the cellular level a stronger response than WT (Supplementary Fig. 15). As shown in Fig. 4e, prolonged treatment with zaxinone led to significant accumulation in the glycosylated, deactivated *trans*-zeatin. Finally, we chose four genes annotated by Kyoto Encyclopedia of Genes and Genomes analysis as cytokinin glycosyltransferases (Supplementary Data 5), and validated their expression levels in the samples used for the RNA-seq experiment, using qRT-PCR. As anticipated, these genes were highly induced following 24 h of zaxinone treatment (Fig. 2c and Supplementary Fig. 18). We obtained similar results in *zas* mutant seedlings exposed to zaxinone treatment (Supplementary Fig. 19). The induction of these genes may explain the accumulation of the glycosylated forms of CKs. To further validate that reduction of cytokinin signaling via CK glycosylation is a part of the downstream effect of zaxinone, we applied 2.5 μM zaxinone to CK biosynthetic (*Os03g49050*, *Os05g51390*, and O*s10g33900*) and regulatory (*Os06g08440*) rice T-DNA insertion mutants (Supplementary Fig. 20a). Interestingly, none of these mutants showed an increased root growth in response to zaxinone treatment, in contrast to the corresponding WT controls (Supplementary Fig. 20b). This result supported the hypothesis that the root growth-promoting effect of zaxinone is dependent on CKs.

## Discussion

**Fig. 5:**
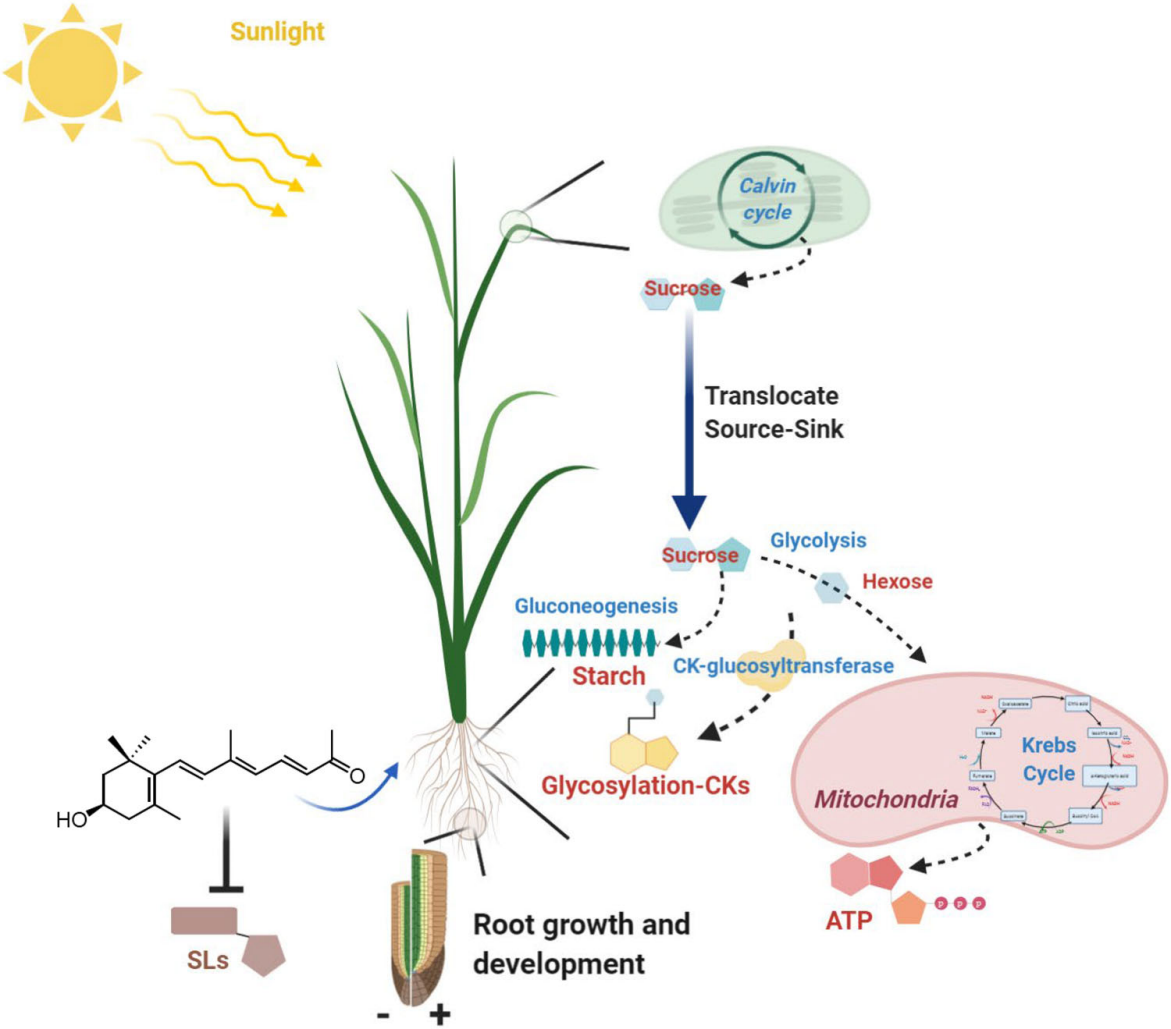
Model of the mechanisms underlying the growth-promoting effect of zaxinone in rice. Application of zaxinone might enhance photosynthesis activity (Calvin cycle) that produces sucrose that is translocated from the shoot (source) to developing root tissues (sink). Sucrose then can either be hydrolyzed (glycolysis) into hexose that enters glycolysis and citric acid cycle (to produce ATP and C-building blocks), or stores as starch formed by gluconeogenesis. The glucose can also be used for cytokinin glycosylation that regulates the bioactivity of CKs in rice root tissues. A combination of these effects results in root phenotypical changes and cellular events, such as a larger meristem size. In addition, zaxinone suppresses SL biosynthesis and release, while it cannot rescue the SL biosynthesis and perception mutants^22^ and does not affect the central metabolism in the SL biosynthetic *d17* mutant. Created with “Biorender”.

As the main product of photosynthetic carbon assimilation, sucrose plays an energy source and an essential role in plant growth and development. In addition, this sugar acts as a signaling molecule interacting with hormonal networks and regulating metabolic pathways^25–27^. In this paper, we show that zaxinone application promotes sugar metabolism in growing plants, leading to the accumulation of soluble sugars in different tissues, and might enhance the photosynthetic activity in rice seedlings (Fig. 5). We observed this effect also in *zas* mutant plants (Supplementary Fig. 5c), which contain less zaxinone in their roots^23^. Compared to WT, these plants also showed a lower chlorophyll content under control conditions, suggesting that endogenous zaxinone level might affect the photosynthetic capacity (Supplementary Fig. 5c). The alterations in shoot metabolism were of considerably lower magnitude and slower in response, compared to those of root metabolism. The alterations in root sugar metabolism appear to be biphasic with an initial accumulation of the major sugars sucrose, glucose, and fructose whose levels subsequently decreased, whilst those of downstream metabolites including trehalose, glucose-1-phosphate, glucose-6-phosphate alongside TCA cycle intermediates increased 24 h following zaxinone treatment. We observed similar metabolic changes to the initial effect also in *zas* mutant plants (Fig. 3a). Yet, we cannot rule out that zaxinone might not directly act as signaling molecules as it may need further modification or trigger its response via unknown component(s), which might explain the low response at the 2 h time point and the delay of metabolic changes the root and shoot tissues. Supporting the metabolomics data, the transcriptomic results indicated that zaxinone induces several sugar-related metabolic pathways, such as glycolysis that catabolizes hexose units to produce energy and building blocks for different cellular components (Fig. 5). We also showed that prolonged treatment with zaxinone led to an increased starch accumulation in roots, which is synthesized from mobilized sucrose that is produced by photosynthesis in leaves^26^. In rice, disruption in sucrose synthesis or transport mutations of rice causes growth retardation^28–30^. It might be a possibility that sucrose provides the energy and C-atoms for the plant to grow and develop, depending on zaxinone, as we saw that zaxinone rescued *Oszas* mutant phenotype^23^. This trend correlates with the observed increase in transcript levels of sucrose metabolic genes. Moreover, we observed some of the sugar metabolites were highly accumulated in *zas* mutant compared to WT at control condition, which may argue that the endogenous zaxinone level is involved in the sugar metabolism. However, *zas* also shows high SL content^22^ that indicates the endogenous relative amount of zaxinone and SLs might contribute to the observed sugar metabolites as well as phenotypic changes.

Roots and young leaves are the major sinks during early developmental stages, whereas fruit and seeds are the ones accumulating starch during the reproductive stages^31^. Consequently, zaxinone treatment, which led to an increased starch accumulation in roots, improved the sink capacity of this organ. However, we cannot currently demonstrate whether the increase in chlorophyll abundance and stomatal conductance is due to an enhanced sink-strength of the zaxinone-treated roots, or a direct effect of zaxinone/derivative thereof. Regardless, our results demonstrate zaxinone remarkably increases sugar metabolism and might modulate photosynthesis in rice plants.

The root system is essential for plants to absorb nutrients and water, which determine plant growth and performance. Application of zaxinone remarkably increased crown root numbers^23^, root apex length as well as cortex layers and cell numbers in both rice WT and *zas* mutant, which indicates a possible interplay with Auxin or CKs. Indeed, CKs orchestrate root growth and development; and previous studies documented that this hormone inhibits root elongation by decreasing root meristem size^32–34^. For example, disruption of *CROWN ROOTLESS5* (*CRL5*) encoding an ERF transcription factor led to a loss of rice crown root initiation through repression of two negative regulators of CK signaling (*OsRR1* and *OsRR2*)^35^. Indeed, we detected significant upregulation of the transcript level of *OsRR6*, (*Os04g57720*) and *OsRR10*, (*Os02g35180*), two homologs of *OsRR1* and *OsRR2*, at 2 h after zaxinone application (Supplementary Data 1). Similarly, overexpressing *CK OXIDASE/DEHYDROGENASE 4* (*OsCKX4*, *Os01g71310*) led to lower amounts of CKs, which was accompanied by longer roots and a larger root apical meristem with more cellular layers^34,36^. In this study, we observed an upregulation of *OsCKX4* expression (2 and 24 h; Supplementary Data 1) upon zaxinone treatment, which may contribute to the increased activity of root meristems and alterations in root architecture. Besides, the plant hormones profile and transcript analysis showed that zaxinone reduced the free-form CKs and enhanced glycosylated-CKs biosynthesis, which can be considered as fine-tuning of their synthesis, metabolism, and function. Indeed, glycosylation was shown to significantly reduce the activity of CKs and to affect their transport, signal transduction, and impact on growth and development^37^. The changes in root architecture caused by CK glycosylation resemble those observed upon zaxinone treatment to CK biosynthesis and signaling mutants. Therefore, CKs-glycosylation is of great significance for understanding the effects of zaxinone and its impact on rice root development (Fig. 5). Previously, we showed that zaxinone did not enhance the root growth in SL-deficient rice mutants, indicating the requirement for intact SL biosynthesis^23^. Here, we further found that zaxinone did not increase the sugar metabolism in the SL-deficient rice *d17* mutant, which indicates that the effect of zaxinone on sugar metabolism is mediated by SLs. Taken together, zaxinone not only modulates the SL biosynthesis and release^23^, but also acts on the CK signaling pathway by modulating CK activity through glycosylation. The latter may be the result of increasing sugar content in the root tissues.

In summary, we provide experimental evidence at the metabolite, transcript, and cellular level, which demonstrates the role of zaxinone in regulating central metabolism, determining hormone profile, and promoting cell division in rice roots (Fig. 5). The results presented explain zaxinone’s growth-promoting effect in rice plants and may help to develop new strategies to increase the performance of this and other crops towards sustainable agriculture.

## Methods

### Plant material and growth conditions

Nipponbare background *zas*^23^, *d17*^38^, and WT rice plants were grown under controlled conditions (a 12 h photoperiod, 200-µmol photons m^−2^ s^−1^ and day/night temperature of 27/25 °C). Rice seeds were surface-sterilized in a 50% sodium hypochlorite solution with 0.01 % Tween-20 for 15 min. The seeds were rinsed with sterile water and germinated in the dark overnight. The pre-germinated seeds were transferred to Petri dishes containing half-strength liquid Murashige and Skoog (MS) medium and incubated in a growth chamber for 7 days. Thereafter, the seedlings were transferred into black falcon tubes filled with half-strength modified Hoagland nutrient solution with adjusted pH to 5.8. The nutrient solution consisted of 5.6 mM NH_4_NO_3_, 0.8 mM MgSO_4_·7H_2_O, 0.8 mM K_2_SO_4_, 0.18 mM FeSO_4_·7H_2_O, 0.18 mM Na_2_EDTA·2H_2_O, 1.6 mM CaCl_2_·2H_2_O, 0.8 mM KNO_3_, 0.023 mM H_3_BO_3_, 0.0045 mM MnCl_2_·4H_2_O, 0.0003 mM CuSO_4_·5H_2_O, 0.0015 mM ZnCl_2_, 0.0001 mM Na_2_MoO_4_·2H_2_O and 0.4 mM K_2_HPO_4_·2H_2_O.

For metabolomic and transcriptomic analysis, 3-weeks-old seedlings were grown hydroponically in half-strength modified Hoagland nutrient solution. Seedlings were further treated with 5 µM zaxinone for 2, 6, or 24 h, and tissues were collected.

For phenomic experiments, one-week-old seedlings were grown hydroponically in half-strength modified Hoagland nutrient solution with or without 5 µM zaxinone for two weeks. Thereafter, plant tissues were collected for analysis.

For zaxinone application to CK mutants, 1-week-old seedling (TN67 background) were grown hydroponically in ½ strength Hoagland nutrient solution containing 2.5 µM zaxinone for 2 weeks. The solution was changed two times per week.

Synthetic zaxinone was purchased (custom synthesis) from Buchem B.V. (Apeldoorn, The Netherlands).

### Analysis of primary metabolites using GC-MS

Frozen ground material, spiked with 60 μg phenyl-β-glucopyranosides, was homogenized in 750 μL of methanol at 70 °C for 15 min and then 375 μL of chloroform followed by adding 750 μL of water. The polar fraction was dried under vacuum, and the residue was derivatized for 40 min at 37 °C (in 50 µL of 20 mg mL^−1^ methoxyamine hydrochloride in pyridine) followed by a 30 min treatment at 37 °C with 70 µL of MSTFA. The GC-MS system used was a gas chromatograph coupled to a time-of-flight mass spectrometer (Leco Pegasus HT TOF-MS). A Gerstel Multi Purpose autosampler system injected the samples. Helium was used as carrier gas at a constant flow rate of 2 mL s^−1^ and gas chromatography was performed on a 30 m DB-35 column. The injection temperature was 230 °C and the transfer line and ion source were set to 250 °C. The initial temperature of the oven (85 °C) increased at a rate of 15 °C min^−1^ up to a final temperature of 360 °C. After a solvent delay of 180 s mass spectra were recorded at 20 scans s^−1^ with *m*/*z* 70–600 scanning range. Chromatograms and mass spectra were evaluated by using Chroma TOF 4.5 (Leco) and TagFinder 4.2 software^39,40^.

### Lipid profile by LC–MS

Lipids were extracted based on the protocol published in^41^. In brief, 5 mg of freeze-dry materiel was homogenized and extracted with 1 mL of pre-cooled (−20 °C) extraction buffer (homogenous methanol/methyl-*tert*-butyl-ether [1:3] mixture). After 10 min incubation at 4 °C and sonication for 10 min in a sonic bath, 500 μL of water/methanol mixture was added. Samples were then centrifuged (5 min, 14,000×*g*), which led to the formation of two phases: a lipophilic phase and a polar phase. Five hundred microliters of the lipophilic phase were collected and dried under vacuum and resuspended in 200 μL of ASN/isopropanol and used for lipid analysis. Samples were processed using UPLC-FT-MS on a C_8_ reverse-phase column (100 × 2.1 mm × 1.7 μm particle size, Waters) at 60 °C. The mobile phases consisted of 1% 1 M NH_4_OAc and 0.1 % acetic acid in water (buffer A) and acetonitrile/isopropanol (7:3, UPLC grade BioSolve) supplemented with 1 M NH_4_Ac and 0.1% acetic acid (buffer B). The dried lipid extracts were resuspended in 500 μL of buffer B. The following gradient profile was applied: 1 min 45% A, 3 min linear gradient from 45% A to 35 % A, 8 min linear gradient from 25 to 11% A, 3 min linear gradient from 11 to 1% A. Finally, after washing the column for 3 min with 1% A, the buffer was set back to 45% A, and the column was re-equilibrated for 4 min, leading to a total run time of 22 min. The flow rate of the mobile phase was 400 μL min^−1^. The mass spectra were acquired using an Exactive mass spectrometer (ThermoFisher, http://www.thermofisher.com) equipped with an ESI interface. All the spectra were recorded using altering full-scan and all-ion fragmentation scan mode, covering a mass range from 100–1500 *m*/*z* at a capillary voltage of 3.0 kV, with a sheath gas flow value of 60 and an auxiliary gas flow of 35. The resolution was set to 10,000 with 10 scans per second, restricting the Orbitrap loading time to a maximum of 100 ms with a target value of 1E6 ions. The capillary temperature was set to 150 °C, while the drying gas in the heated electrospray source was set to 350 °C. The skimmer voltage was held at 25 V, while the tube lens was set to a value of 130 V. The spectra were recorded from minute 1 to 20 of the UPLC gradients. Processing of chromatograms, peak detection, and integration was performed using REFINER MS 10.0 (GeneData, http://www.genedata.com) or Xcalibur (Version 3.1, ThermoFisher, Bremen, Germany). In the first approach, the molecular masses, retention time, and associated peak intensities for the three replicates of each sample were extracted from the raw files, which contained the full-scan MS and the all-ion fragmentation MS Data Processing of MS data included the removal of the fragmentation information, isotopic peaks, and chemical noise. Further peak filtering on the manually extracted spectra or the aligned data matrices was performed. Obtained features (*m*/*z* at a certain retention time) were queried against an in-house lipid database for further annotation

### RNA library preparation and transcriptomic analysis

Total rice root RNA was extracted with TRIzol (Invitrogen, https://www.thermofisher.com/de/de/home.htmL) using a Direct-zol RNA Miniprep Plus Kit following the manufacturer’s instructions (ZYMO RESEARCH; USA). RNA quality was checked with a Agilent 2100 Bioanalyzer, and RNA concentration was measured using a Qubit 3.0 Fluorometer. The cDNA libraries were constructed following standard Illumina protocols and paired‐end sequenced on an Illumina HiSeq 4000 machine by the Bioscience core lab of KAUST. RNA‐Seq reads were aligned to the *O. sativa* genome v7.0 downloaded from Phytozome v12.1 (http://phytozome.jgi.doe.gov/). Data processing and analysis were performed using the LSTrAP workflow^42^, which included all steps described below. Adapter sequences were removed from fastq files by Trimmomatic^43^, and aligned to the genome using HISAT2^44^. Read counts aligned to each annotated gene were computed with HTSeq^45^. The results were passed through LSTrAP quality control and TPM normalized. The mean data were used to cluster and resistance level was visualized as a heatmap using a hierarchical clustering R script. Principal component analysis (PCA), a multivariate statistical technique, was further conducted to examine links between samples. All analyses were performed using the R statistical package. For differential gene expression, read counts from HTSeq were analyzed using the R package DESeq2^46^. Genes were considered differentially expressed based on a *P*‐value adjusted by the Benjamini–Hochberg procedure^47^ below 0.05. Gene Ontology (GO) enrichment analysis of all the differentially were selected at FDR < 0.05 and analyzed by Panther-Gene list analysis^48^ (http://pantherdb.org/). Kyoto Encyclopedia of Genes and Genomes and PlantCyc pathway enrichment analysis for up- and downregulated genes, were then analyzed by The Plant GeneSet Enrichment Analysis Toolkit (PlantGSEA)^49^ (http://structuralbiology.cau.edu.cn/PlantGSEA/index.php). Visualization of DEGs in MapMan followed the instructions as described^50^.

### Gene expression analysis

Roots of rice seedlings were ground and homogenized in liquid nitrogen, and total RNA was isolated using a Direct-zol RNA Miniprep Plus Kit following the manufacturer’s instructions (ZYMO RESEARCH; USA). cDNA was synthesized from 1 µg of total RNA using iScript cDNA Synthesis Kit (BIO-RAD Laboratories, Inc, 2000 Alfred Nobel Drive, Hercules, CA; USA), according to the instructions in the user manual. Transcript levels were detected by real-time quantitative RT-PCR (qRT-PCR) which was performed using SYBR Green Master Mix (Applied Biosystems; www.lifetechnologies.com) in a CFX384 Touch™ Real-Time PCR Detection System (BIO-RAD Laboratories, Inc, 2000 Alfred Nobel Drive, Hercules, CA; USA). Primers used for qRT-PCR analysis are listed in Supplementary Data 8. The gene expression level was calculated by normalization to the rice housekeeping gene Ubiquitin (OsUBQ) (Supplementary Data 8). The relative gene expression level was calculated according to 2^−ΔΔ^CT method^51^.

### Cotton blue staining for root apex

Apex length and width were assessed in WT rice roots with or without 5 μM zaxinone (applied twice a week). Plants were grown hydroponically in Hoagland solution (400 µM Pi), and data were collected 3 weeks post-germination. The primary crown root apex was stained with Cotton Blue 0.1 %. The apex length was calculated considering the segment between the root tip and the first root hair.

### Ethynyl deoxyuridine (EdU) staining for cell proliferation analysis

Cell proliferation in rice seedlings was evaluated using the Click-iT EdU Alexa Fluor 488 imaging kit (C10637, Invitrogen) followed the procedure^52^. Plants were incubated in Murashige and Skoog medium with EdU for 12 h. For each plant, the primary root and the longest crown root (prior to the formation of lateral roots) were harvested and fixed in 3.7% formaldehyde for 1 h under vacuum. Then samples were permeabilized with PBS containing 0.5 % Triton X-100 for 1 h and incubated for 1 h in the dark with a click-it-reaction cocktail that was prepared according to the manual, followed by DNA counterstaining using Hoechst 33342 in PBS under vacuum in the dark for 1 h. Samples were mounted in clearing solution and incubated in the dark for 2 weeks at 4 °C as described in the protocol published in ref. ^53^. Images were captured by a Zeiss LSM 880 inverted confocal microscope and automatically stitched to generate the overview image of the root tip in ZEN 2.0 while imaging. Dividing EdU-stained nuclei are shown in green; nuclei counterstained with Hoechst 33258 are shown in blue. Images are representative of the total number (*n* = 10) of seedlings that were studied.

### Root cross section, staining, and microscopy

Fresh root segments starting from the root hair emergence zone to the direction of the shoot (an upward direction, about 0.5 cm from in differentiation zone) were embedded in 10% low melting agarose and sectioned using a Leica VT1000S vibratome. The SCRI Renaissance 2200 (SR2200) stain was used to visualize cell walls while the berberine hemisulfate stain was used to visualize suberin^54^. Images were captured using a Zeiss LSM 880 inverted confocal microscope with excitation of 405 nm for SCRI or 488 nm for berberine.

### Quantification of starch

For starch extraction, excised root systems were rapidly blot-dried on filter paper and weighed. Samples were then frozen in liquid nitrogen, transferred to 2-mL Eppendorf tubes (Eppendorf, Hamburg, Germany), and thoroughly homogenized using a pestle in liquid nitrogen. The samples were further homogenized in 0.5 mL of absolute ethanol. After the addition of 0.5 mL of 80% ethanol, the tubes were incubated at 70 °C for 90 min and then centrifuged for 10 min at 11,337 × *g* and the pellet was resuspended in 1 mL of 80% ethanol. Two more washings were performed with 1 mL of 80% ethanol (and 10 min of centrifugation). The pellets were finally resuspended in 400 μL of 0.2 m KOH and incubated at 95 °C for 60 min. After neutralization with 70 μL of acetic acid, the samples were centrifuged for 10 min and the supernatant was used for starch quantification (Starch Test-Combination enzymatic analysis kit, cat. no. 207748; Boehringer, Mannheim, Germany), according to the manufacturer’s instructions. At least three independent experiments, including at least three plants each, were performed to obtain all results of enzymatic starch quantification.

### Quantification of plant hormones

For the quantification of endogenous hormone levels, 20 mg of freeze-dried ground tissues were spiked with internal standards D_6_-ABA (3.2 ng), D_2_-GA1 (0.08 ng), D_2_-IAA (5.4 ng), D_4_-SA (0.05 ng), D_2_-JA (0.1 ng), D_5_-*trans*-zeatin (1.5 ng), D_5_-*trans*-zeatin-O-glucoside (2 ng), D_5_-*trans*-zeatin riboside-O-glucoside (2 ng), D_6_-N^6^-Isopentenyladenine (2 ng), N_15_-N^6^-isopentenyladenosine (2 ng), and D_7_-N^6^-Benzyladenine (2 ng) along with 1.5 mL of methanol as described procedure^51^. The mixture was sonicated for 15 min in an ultrasonic bath (Branson 3510 ultrasonic bath), followed by centrifugation for 10 min at 14,000 × *g* at 4 °C. The supernatant was collected, and the pellet was re-extracted with 1.5 mL of the same solvent. Then, the two supernatants were combined and dried under a vacuum. The sample was re-dissolved in 150 μL of acetonitrile:water (25:75, v-v) and filtered through a 0.22 μm filter for LC–MS analysis. Plant hormones were analyzed using HPLC-Q-Trap-MS/MS with Multiple Reaction Monitoring (MRM) mode. Chromatographic separation was achieved on a ZORBAX Eclipse plus C_18_ column (150 × 2.1 mm; 3.5 μm; Agilent). Mobile phases consisted of water:acetonitrile (95:5, v-v) and acetonitrile, both containing 0.1% formic acid. A linear gradient was optimized as follows (flow rate, 0.4 mL min^−1^): 0 − 17 min, 10–100% B, followed by washing with 100% B and equilibration with 10% B. The injection volume was 5 μL and the column temperature was maintained at 40 °C for each run. Mass spectrometry was conducted in electrospray and MRM mode, in positive ion mode for cytokinins, and in negative ion mode for the other hormones. Relevant instrumental parameters were set as follows: ion source of turbo spray, ion spray voltage of (±) 4500 V, curtain gas of 25 psi, collision gas of medium, gas 1 of 45 psi, gas 2 of 30 psi, turbo gas temperature of 500 °C, entrance potential of −10 V. The characteristic MRM transitions (precursor ion → product ion) were 263.2 → 153.1 for ABA; 347.1 → 261.1 for GA1; 174.0 → 129.6 for IAA; 136.6 → 92.8 for SA; 209.0 → 59.0 for JA; 269.2 → 159.1 for D_6_-ABA; 349.1 → 261.1 for D_2_-GA1; 176.0 → 131.6 for D_2_-IAA; 141.0 → 97.0 for D_4_-SA ; 211.0 → 61.0 for D_2_-JA. 225.2 → 136.7 for D_5_-*trans*-zeatin; 387.2 → 225.4 for D_5_-*trans*-zeatin- *O* -glucoside; 519.2 → 225.2 for D_5_-*trans*-zeatin riboside-*O*-glucoside; 210.2 → 137.0 for D_6_-N^6^-Isopentenyladenine; 337.0 → 205.0 for N_15_-N^6^-isopentenyladenosine; 233.1 → 98.0 for D_7_-N^6^-Benzyladenine; 220.2 → 136.0 for *trans*-zeatin; 382.2 → 220.2 for *trans*-zeatin-*O*-glucoside; 514.2 → 220.2 for *trans*-zeatin riboside-*O*-glucoside; 204.2 → 136.1 N^6^-Isopentenyladenine; 336.0 → 204.0 for N^6^-isopentenyladenosine; 226.1 → 91.0 for N^6^-Benzyladenine.

### Measurement of photosynthetic parameters

Three-week-old seedlings were grown hydroponically in a half-strength-modified Hoagland nutrient solution. Seedlings were further treated with 5 µM zaxinone for 2, 6, or 24 h. Leaf chlorophyll content was measured by CCM-200 plus chlorophyll content meter (Opti-Sciences, Hudson, USA), and leaf stomatal conductance was measured by AP4 Porometer (Delta-T, Cambridge, UK).

### Chlorophyll quantification

Chlorophyll was extracted from the leaf segment by following the procedure according to ref. ^55^ with a slight modification. Briefly, an equal amount of frozen leaf tissue was measured in a 2 mL Eppendorf tube and ground into a fine powder with 2 mm metal beads. One mL of 80% acetone was added to each tube and the mixture was vortex for 30 s. The extracted mixture was incubated at room temperature for 10 min. Each sample was subjected to centrifugation at 4282 × *g*, 4, at room temperature for 90 s. Then 200 µL of supernatant was collected from the top of each tube and added to 96 well plates. The plate was run in the microplate reader (Tecan Infinite M1000 Pro). The absorbance of Chlorophyll-*a* (Chla) and Chlorophyll-*b* (Chlb) was determined by UV-spectrophotometry at 645 and 663 nm wavelength. Chlorophyll-*a*, Chlorophyll-*b*, and total chlorophyll content were calculated from each extract by using the following equations:

1 $${{{{{\rm{Chl}}}}}}a\,({{{{{\rm{mg}}}}}}\;{{{{{\rm{g}}}}}}^{-1})=12.7({{{{{\rm{A663}}}}}})-2.69({{{{{\rm{A645}}}}}})\times{{V}}/{{{{{\rm{1000}}}}}}\times W$$

2 $${{{{{\rm{Chl}}}}}}b\,({{{{{\rm{mg}}}}}}\;{{{{{\rm{g}}}}}}^{-1})=22.9({{{{{\rm{A645}}}}}})-4.68({{{{{\rm{A663}}}}}})\times{{V}}/{{{{{\rm{1000}}}}}}\times W$$

3 $${{{{{\rm{Total}}}}}}\,{{{{{\rm{Chl}}}}}}\,({{{{{\rm{mg}}}}}}\;{{{{{\rm{g}}}}}}^{-1})=20.2({{{{{\rm{A645}}}}}})+8.02({{{{{\rm{A663}}}}}})\times{{V}}/{{{{{\rm{1000}}}}}}\times W$$

where

*A* = absorbance at specific wavelengths

*V* = final volume of chlorophyll extract

*W* = fresh weight of tissue extracted

### Statistics and reproducibility

A minimum of three independent biological replicates to ensure reproducibility in all the experiments. Exact biological samples (*n*) and mean with error bars are indicated in individual figure captions and methods. Statistical tests were carried out through one-way analysis of variance (one-way ANOVA) and Tukey’s post hoc test or two-tailed Student’s *t*-tests, using a probability level of *P* < 0.05, which was considered to be statistically significant.

### Reporting summary

Further information on research design is available in the Nature Research Reporting Summary linked to this article.

## Supplementary information

Supplemental Information

Description of Additional Supplementary Files

Supplementary Data 1

Supplementary Data 2

Supplementary Data 3

Supplementary Data 4

Supplementary Data 5

Supplementary Data 6

Supplementary Data 7

Supplementary Data 8

Supplementary Data 9

Supplementary Data 10

Supplementary Data 11

Reporting Summary

## Data Availability

All data generated or analyzed during this study are included in this published article, Supplementary Information, and Supplementary Data 10 and 11. RNA-Seq data can be accessed at NCBI’s Gene Expression Omnibus (GEO) via accession number (GSE184529).
