# Supplementary material for: Multi-omics approaches explain the growth-promoting effect of the apocarotenoid growth regulator zaxinone in rice
Source: Commun Biol. 2021 Oct 25;4:1222. doi: 10.1038/s42003-021-02740-8 (PMC8545949; doi:10.1038/s42003-021-02740-8)
Supplement: Supplementary file 1 — Supplemental Information [file 42003_2021_2740_MOESM1_ESM.pdf]

Supplementary Information

Multi-Omics Approaches Explain the Growth-Promoting Effect of the Apocarotenoid Regulator Zaxinone in Rice

Jian You Wang, Saleh Alseekh, Tingting Xiao, Abdugaffor Ablazov, Leonardo Perez de Souza, Valentina Fiorilli, Marita Anggarani, Pei-Yu Lin, Cristina Votta, Mara Novero, Muhammad Jamil, Luisa Lanfranco, Yue-Ie C. Hsing, Ikram Blilou, Alisdair R. Fernie, and Salim Al-Babili*

*Correspondence to: salim.babili@kaust.edu.sa.

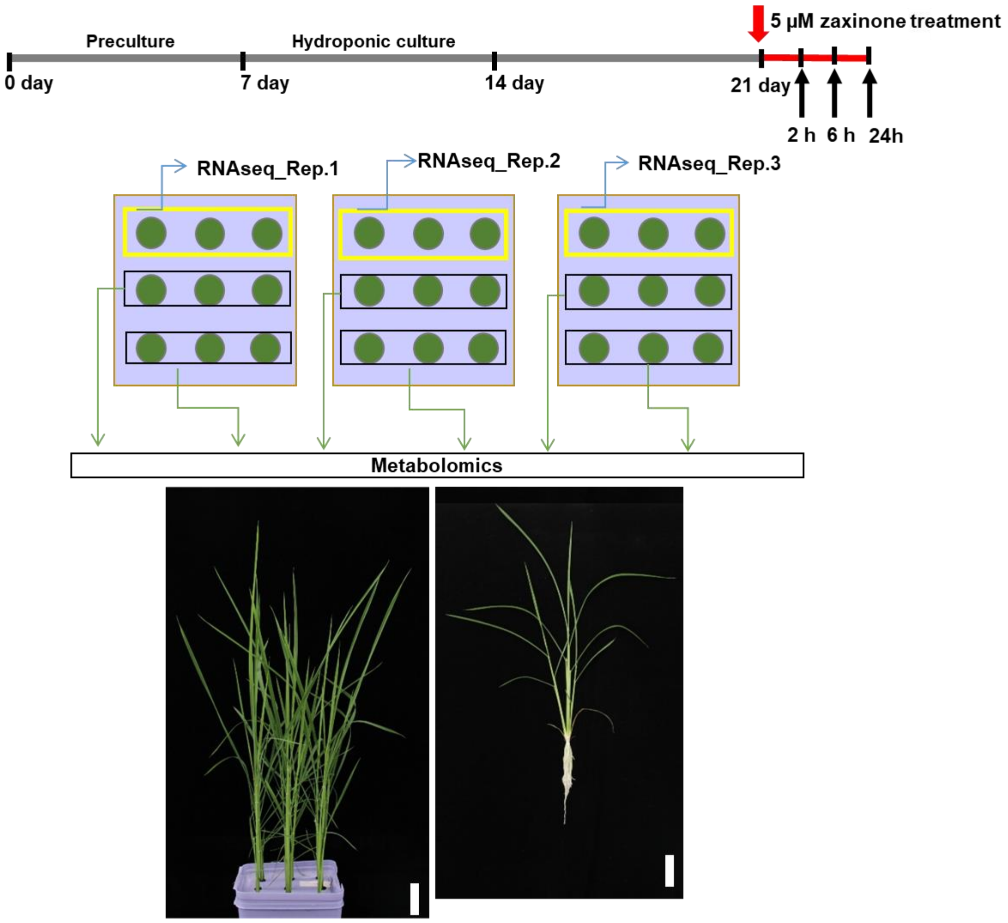

Supplementary Figure 1 Hydroponic system set-up for omics experiments.

Rice plants were grown with half-strength modified Hoagland nutrient solution with adjusted pH to 5.8 for two weeks. Plants were treated with 5 µM zaxinone or 0.1 % Acetone as control at day 21, and thereafter harvested as shown at 2 h, 6 h, and 24 h respectively for metabolomics and transcriptomic analysis. Represented pictures were shown below; scale bar: 5 cm.

a

b

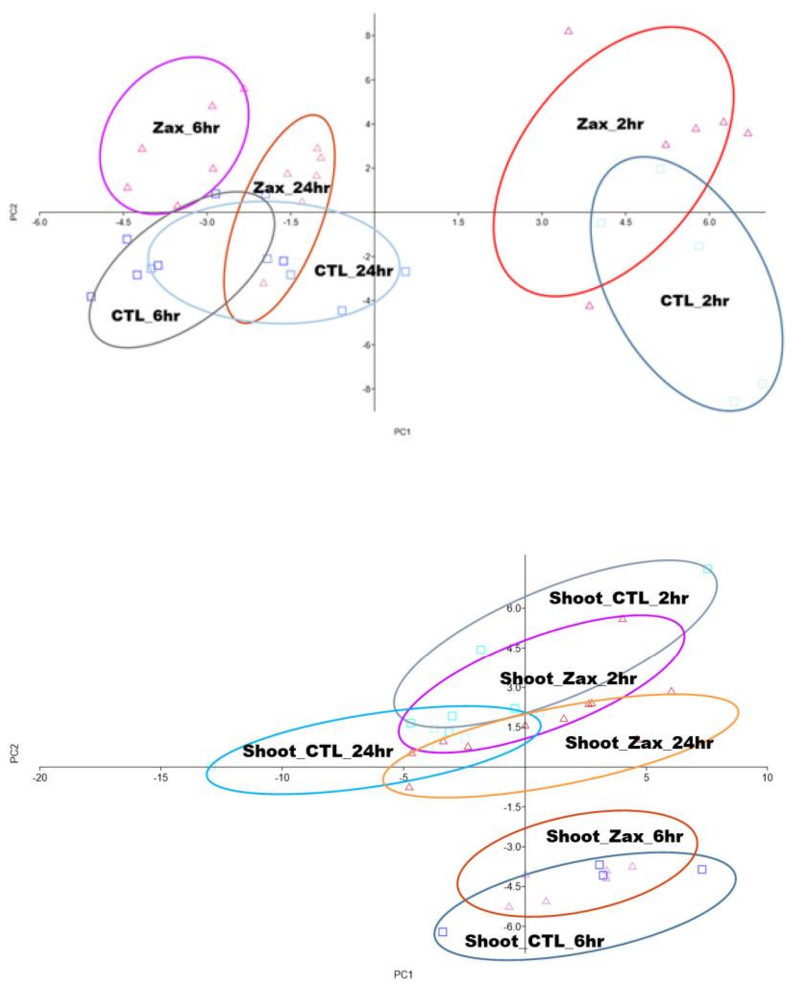

Supplementary Figure 2 Principal component analysis (PCA) of primary metabolites.

a root and **b** shoot tissues**.** Data were analyzed using Past3 software.

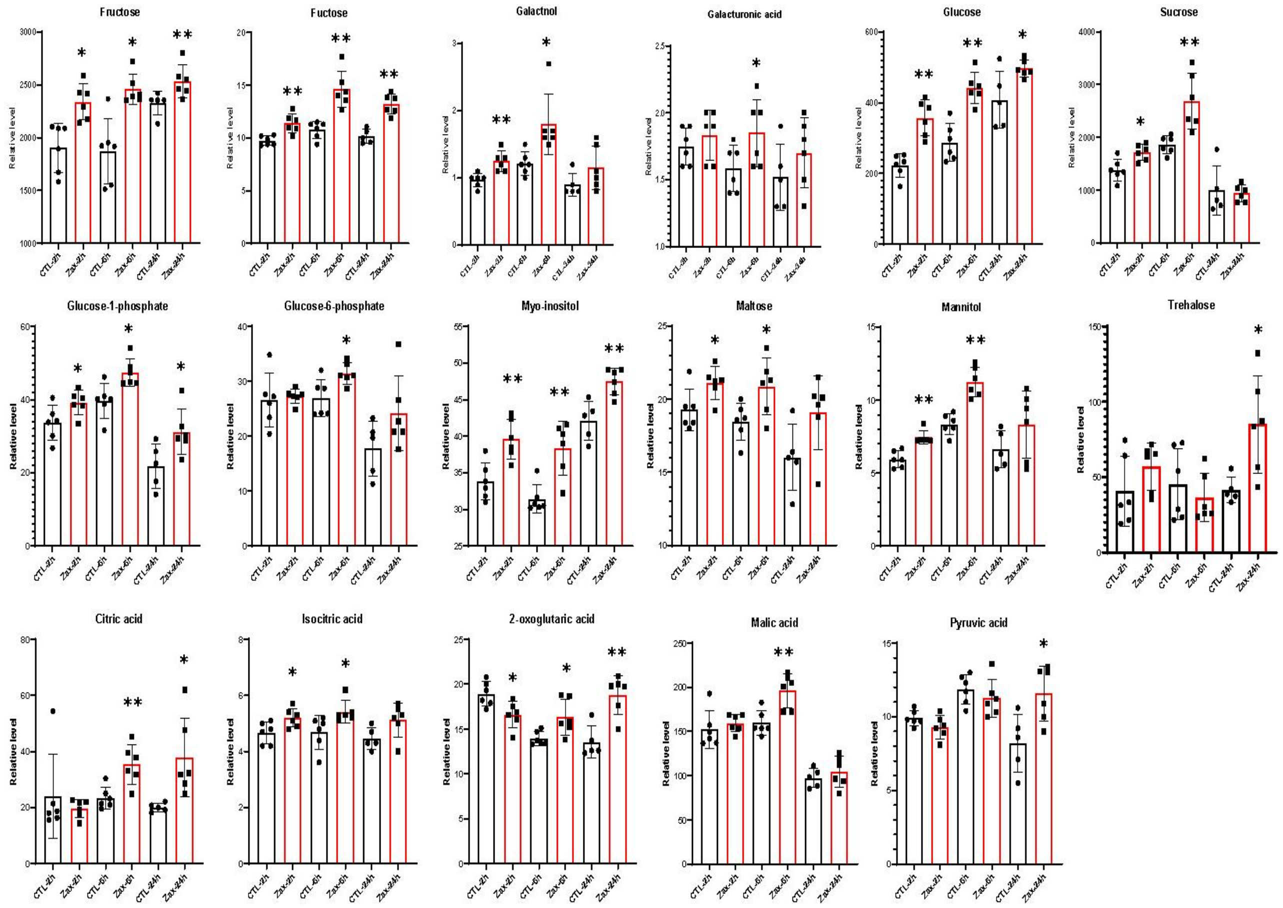

Supplementary Figure 3 Relative level of sugar-related metabolites in root tissues.

Primary metabolites extracted from roots for GC-MS, which were annotated and listed in Supplementary Data 6. Bar represents mean ± SD, *n*≥4 biological replicates. Asterisks indicate statistically significant differences as compared to control by *t*-test (**p*< 0.05, ***p* < 0.01). CTL, control; Zax, zaxinone.

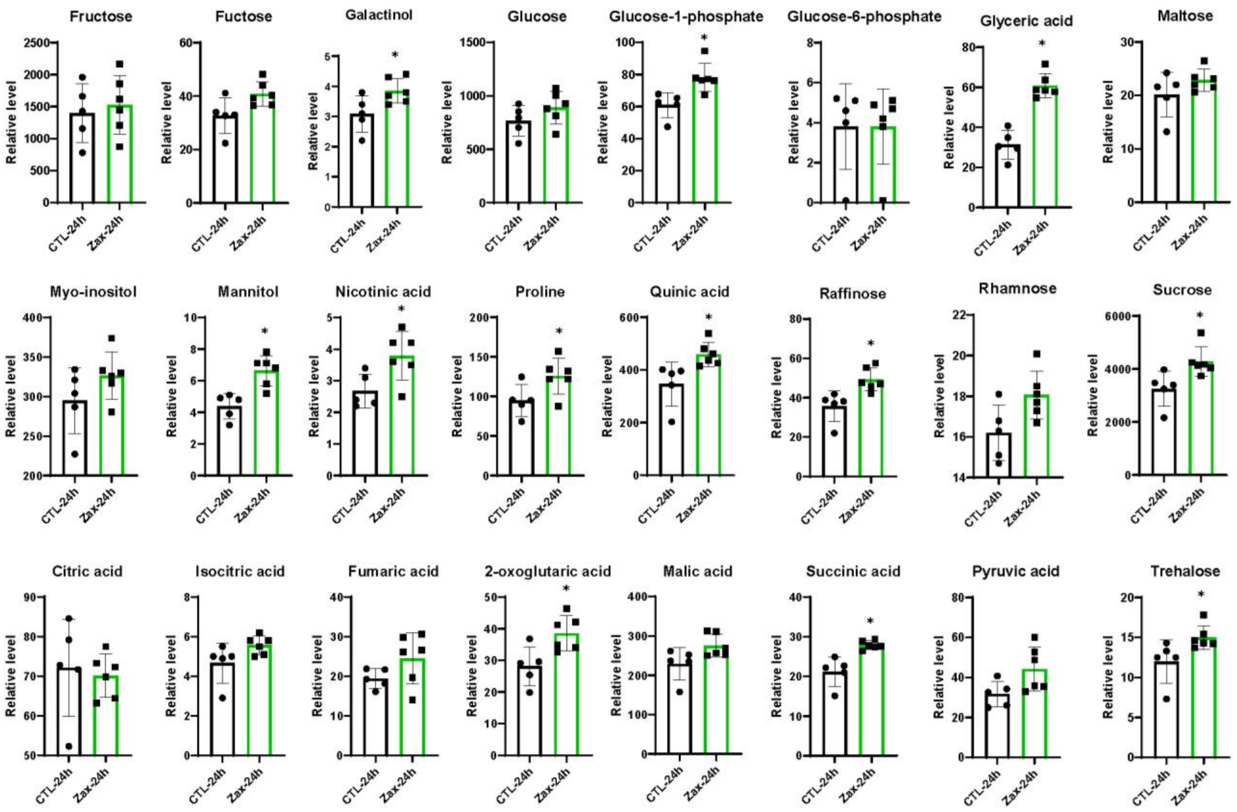

Supplementary Figure 4 Relative level of sugar-related metabolites in shoot tissues.

Primary metabolites extracted from roots for GC-MS, which were annotated and listed in Supplementary Data 6. Bar represents mean ± SD, $n \geq 4$ biological replicates. Asterisks indicate statistically significant differences as compared to control by *t*-test (*$p < 0.05$). CTL, control; Zax, zaxinone.

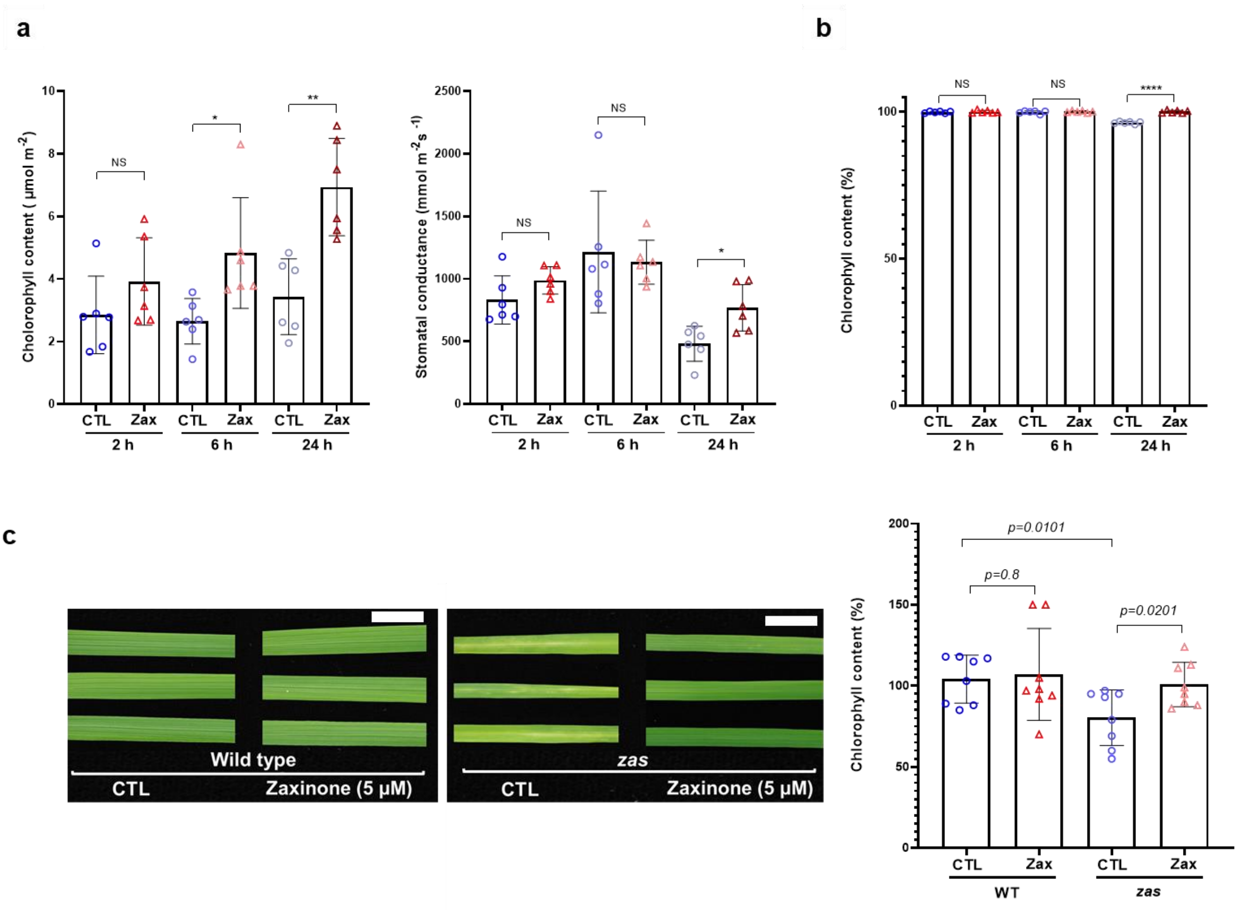

Supplementary Figure 5 Zaxinone enhanced photosynthetic activity.

a Leaf Chlorophyll content: Measured by CCM-200 plus chlorophyll content meter (Opti-Sciences, Hudson, USA). Leaf stomatal conductance: Measured by AP4 Porometer (Delta-T, Cambridge, UK). **b** Chlorophylls were extracted by 80% acetone, and their levels were determined by UV-photometer. *n*=6 biological replicates. **c** Chlorophyll level in shoot tissues of WT and *zas* mutant after 2-week 5 μM zaxinone treatment, *n*=8 biological replicates. Bar represents mean ± SD. Scale bar: 1 cm. Asterisks indicate statistically significant differences as compared to control by *t*-test (*$p < 0.05$, **$p < 0.01$, ***$p < 0.001$, **** $p < 0.0001$). CTL, control; Zax, zaxinone.

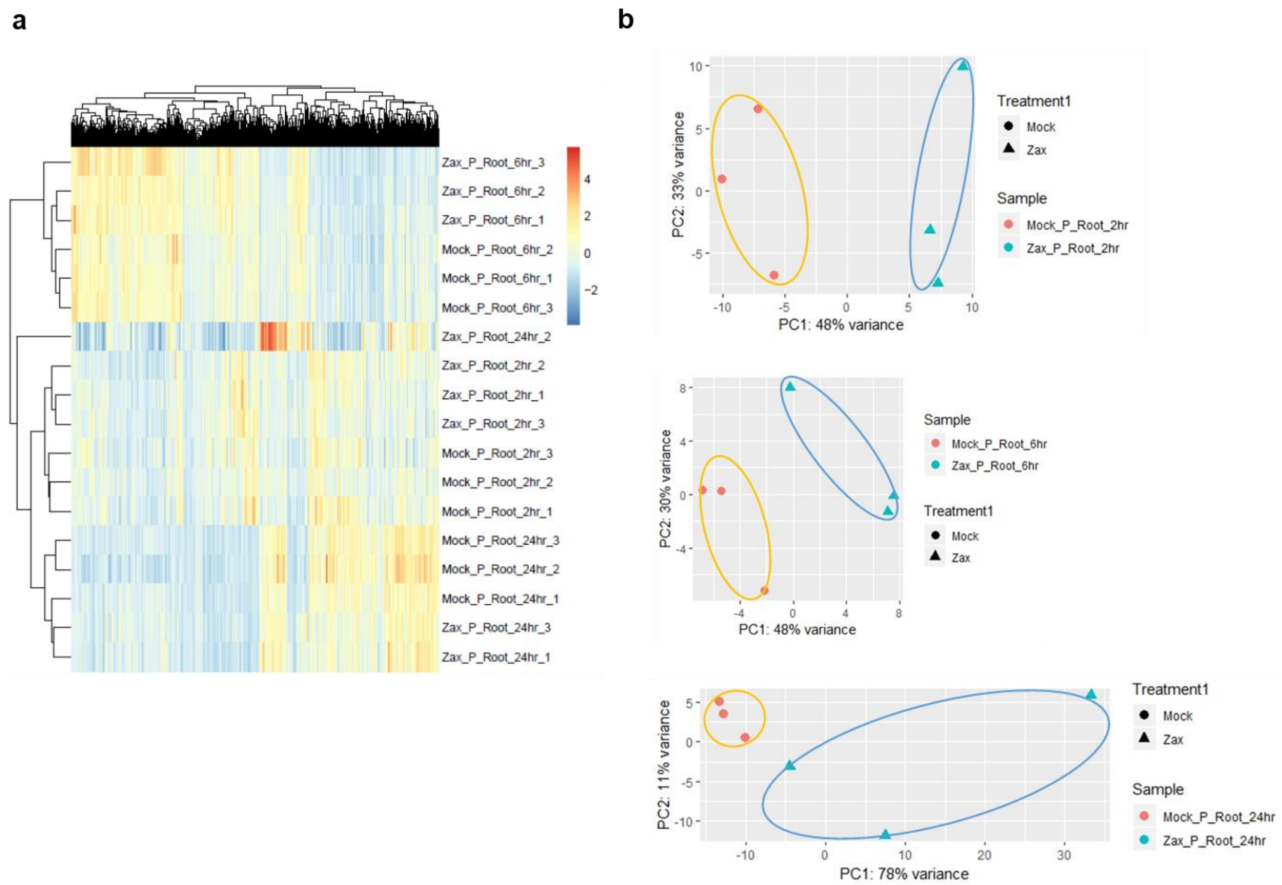

Supplementary Figure 6 Transcriptomic analysis by using Large-Scale Transcriptome Analysis Pipeline (LSTrAP) of root samples.

a Heatmap visualization of mean-centered normalized log-expression values for correlated highly variable genes (HVGs). Color represented as log-transformed of TPM (Transcripts Per Kilobase Million) expression data. **b** Principal component analysis (PCA) plots of HVGs at 2, 6, and 24 hours, respectively. *n*=3 biological replicates.

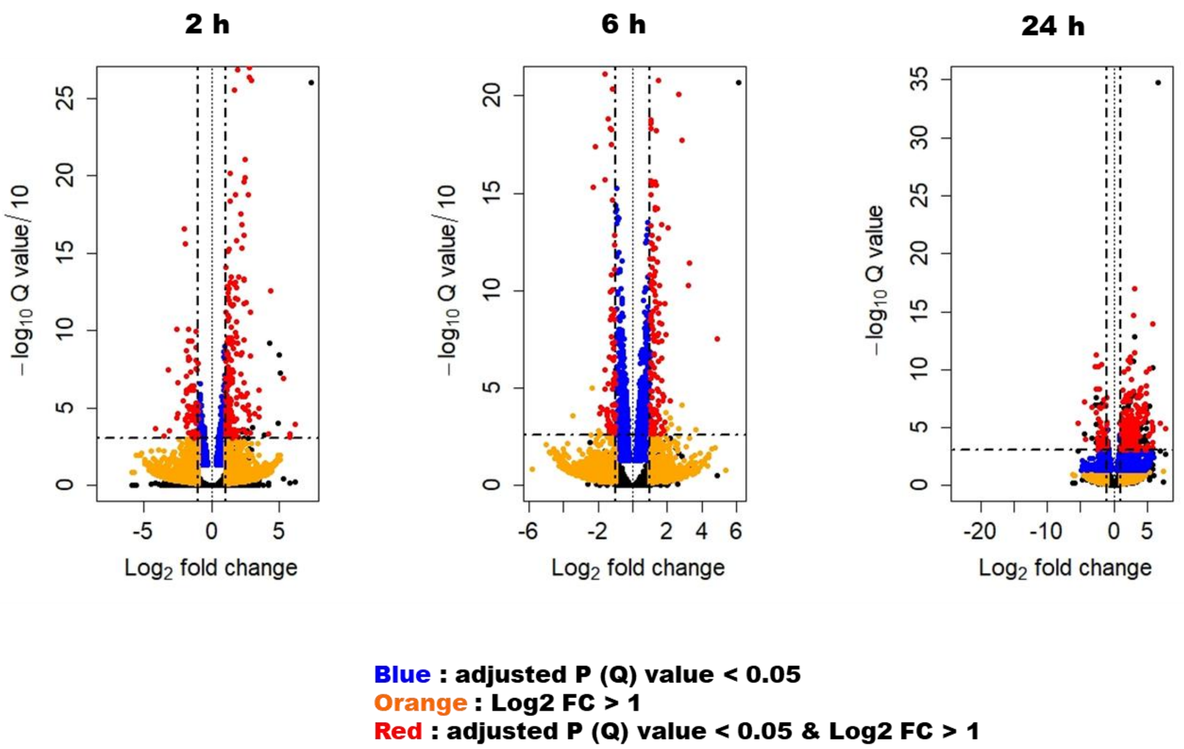

Supplementary Figure 7 Summary of the RNA-Seq results.

Volcano plot representation of differential expression analysis of genes in the control versus zaxinone-treatment data sets. Blue points mark the genes with significantly expression (adjusted P (Q) value, FDR<0.05), orange points represent log^2fold-changes>1, and the red points marks co-existing genes in blue and orange, respectively. Black vertical lines highlight log fold changes of −1 and 1, while Black horizontal line represents a FDR of 0.05.

MSU_Locus	2 h RNAseq (Log2FC)	2 h q-PCR (Log_2FC)	6 h RNAseq (Log2FC)	6 h q-PCR (Log_2FC)	24 h RNAseq (Log2FC)	24 h q-PCR (Log_2FC)
Os01g05150.1	2.85	1.05	1.19	2.07	1.90	1.82
Os01g06660.1	0.63	1.62	0.70	2.15	0.97	2.22
Os01g16170.1	-0.90	-0.32	-0.88	-0.24	-1.20	-0.24
Os01g33869.1	-1.03	-0.58	-0.49	-0.23	-1.32	-0.60
Os10g07556.1	-1.41	-0.96	-0.99	0.21	-1.32	-0.21
Os06g05990.1	-0.67	-0.40	-0.77	-0.07	-0.63	-0.46
Os06g11130.1	1.80	1.95	0.76	1.09	1.65	0.99
Os07g14470.1	-1.89	-1.05	-1.22	-0.91	-2.71	-2.11
Os07g23570.1	5.07	4.83	4.90	3.23	5.87	4.77
Os07g28250.1	-2.61	-0.89	-0.77	-0.15	-1.74	-1.00
Os07g44110.1	1.91	2.89	1.29	2.71	2.06	3.40
Os10g10540.1	-1.09	-0.39	-1.21	0.32	-2.37	-1.48
Os01g42370.1	7.30	7.43	6.13	4.97	6.52	5.08
Os05g45230.1	1.28	1.28	1.09	1.53	1.94	1.48
Os02g35490.1	-0.77	-0.40	-0.82	0.01	-1.18	-0.38

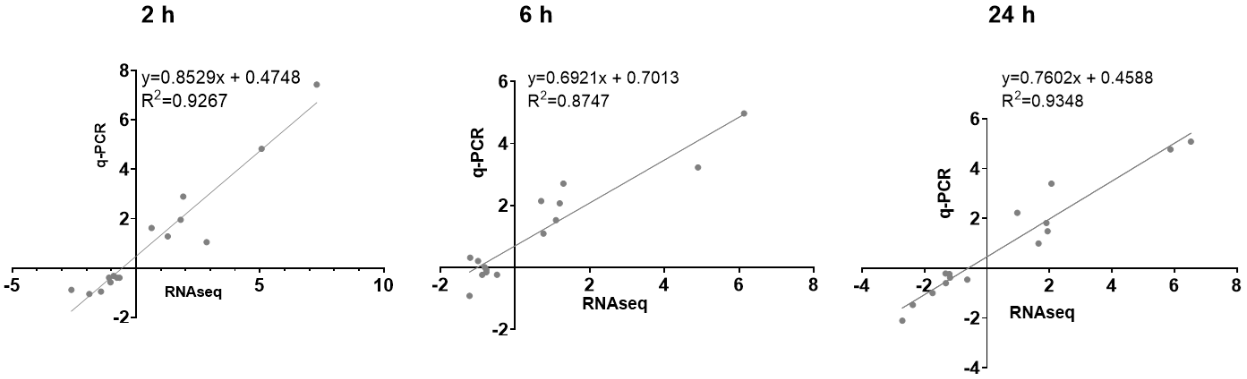

Supplementary Figure 8 Validation of RNAseq by q-RTPCR.

15 selected genes showing high to low fold change in expression upon zaxinone treatment. The resulting correlation analysis (R^2) shown from 0.87-0.93. *n*=3 biological replicates.

a

b

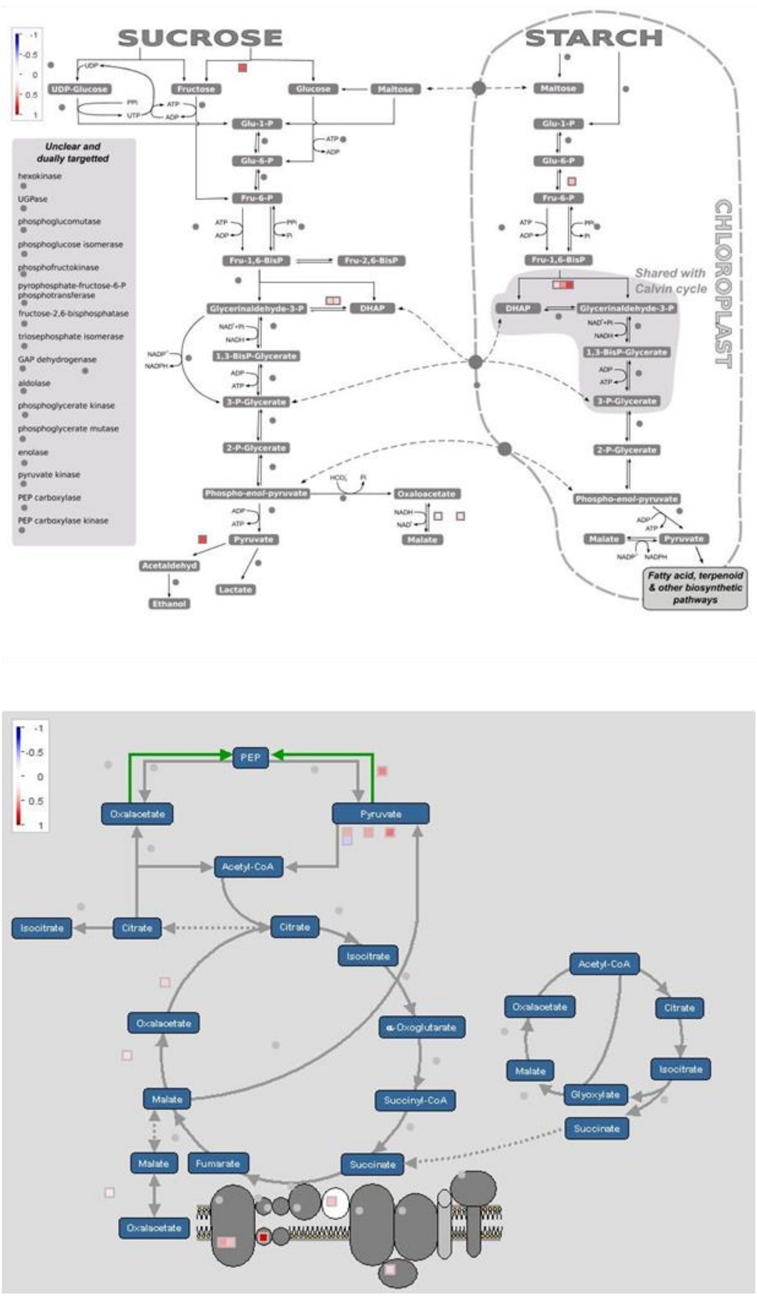

Supplementary Figure 9 MapMan visualization of differentially expressed genes (DEGs) in zaxinone-treated root tissues at 6 hr.

a Plant glycolysis pathway. **b** TCA cycle with electron transport in mitochondria. Fold changes are indicated by color, squares in red denotes up-regulation and blue down-regulation.

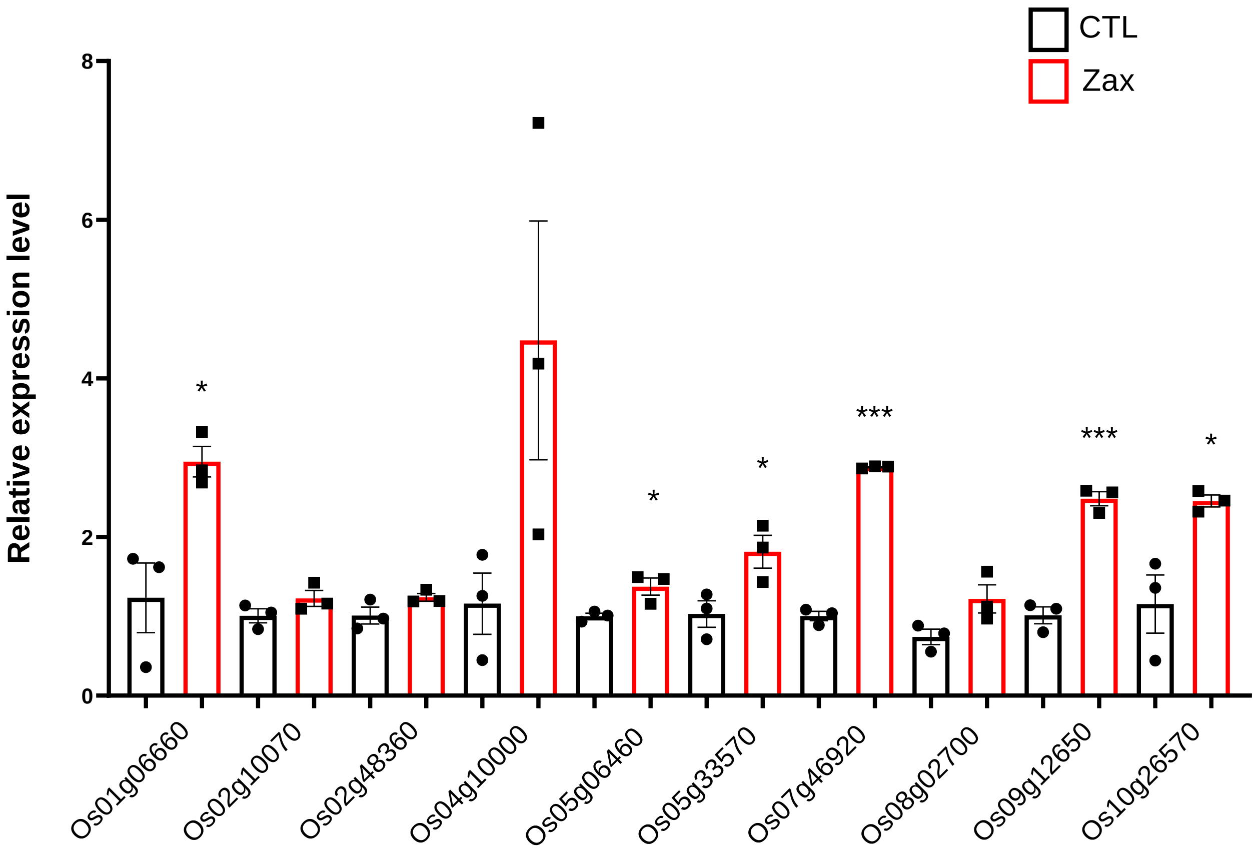

Supplementary Figure 10 Validation of sugar metabolism genes by q-RTPCR in WT plant roots. Ten genes identified from the OyzaCyc 6.0 database, which are involved in glycolysis in the root tissue (Supplementary Data 3), and validated using q-RT-PCR. Bar presented mean ± SEM, n=3 biological replicates. Asterisks indicate statistically significant differences as compared to control by t-test (*$p < 0.05$, **$p < 0.01$, ***$p < 0.001$). CTL, control; Zax, zaxinone.

a b

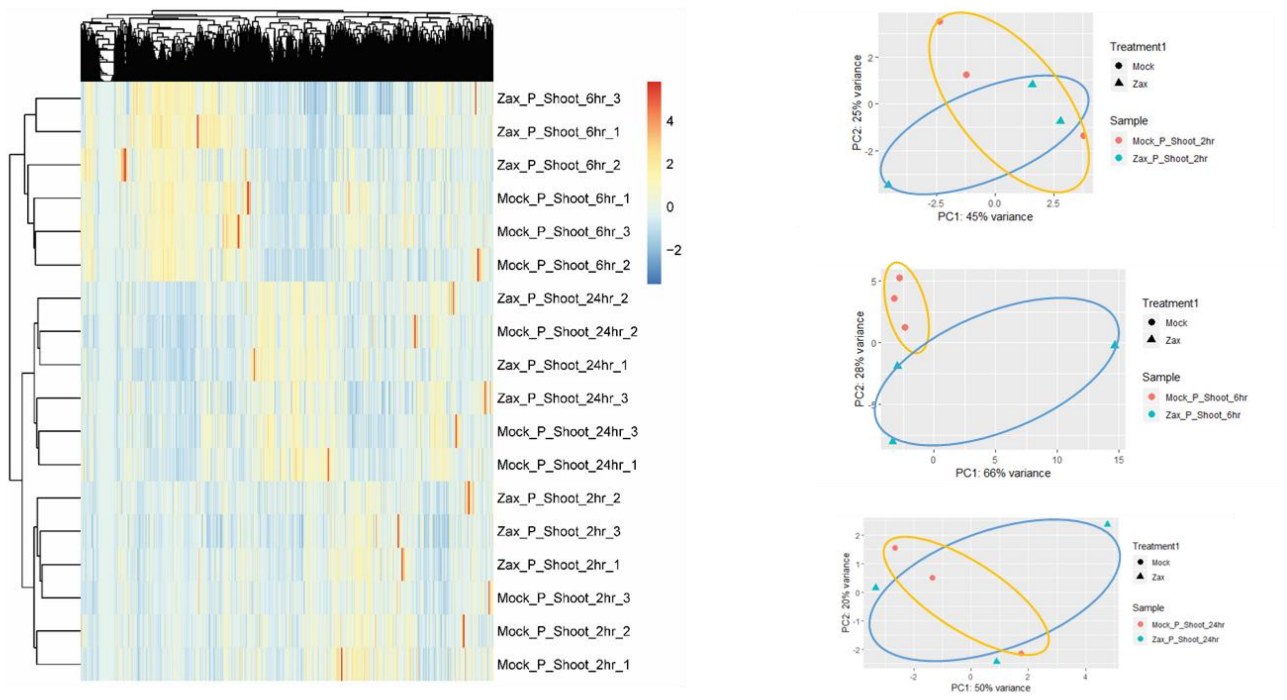

Supplementary Figure 11 Transcriptomic analysis by using Large-Scale Transcriptome Analysis Pipeline (LSTrAP) of shoot samples.

a Heatmap visualization of mean-centered normalized log-expression values for correlated highly variable genes (HVGs). Color represented as log-transformed of TPM (Transcripts Per Kilobase Million) expression data. **b** Principal component analysis (PCA) plots of HVGs at 2, 6, and 24 hours, respectively. *n*=3 biological replicates.

a

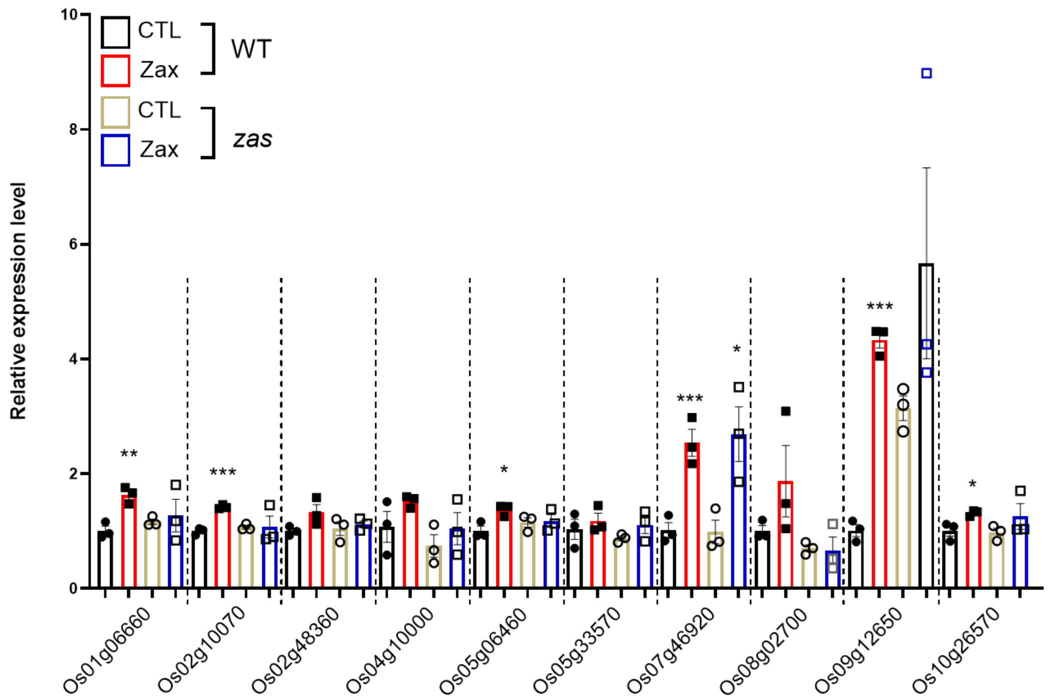

b

Log2FC	Os01g06660	Os02g10070	Os02g48360	Os04g10000	Os05g06460	Os05g33570	Os07g46920	Os08g02700	Os09g12650	Os10g26570
Fig S10 WT	1.26	0.28	0.29	1.95	0.46	0.82	1.52	0.90	1.30	1.09
Fig S12 WT	0.70	0.51	0.40	0.79	0.45	0.20	1.32	0.89	2.10	0.38
Fig S12 *zas*	0.13	0.01	0.10	0.49	0.03	0.34	1.45	-0.08	0.85	0.37

Supplementary Figures. 12 Transcript analysis of root tissues upon 6 h zaxinone treatment in WT and *zas* mutant.

a. Ten genes identified from the OyzaCyc 6.0 database, which are involved in glycolysis in the root tissue (Supplementary Data 3). Bar present mean ± SEM, *n*=3 biological replicates. Asterisks indicate statistically significant differences as compared to control by *t*-test (*$p < 0.05$, **$p < 0.01$, ***$p < 0.001$). CTL, control; Zax, zaxinone. **b**. Heatmap analysis revealed the up-regulating correlation of WT and *zas* mutant after zaxinone treatment.

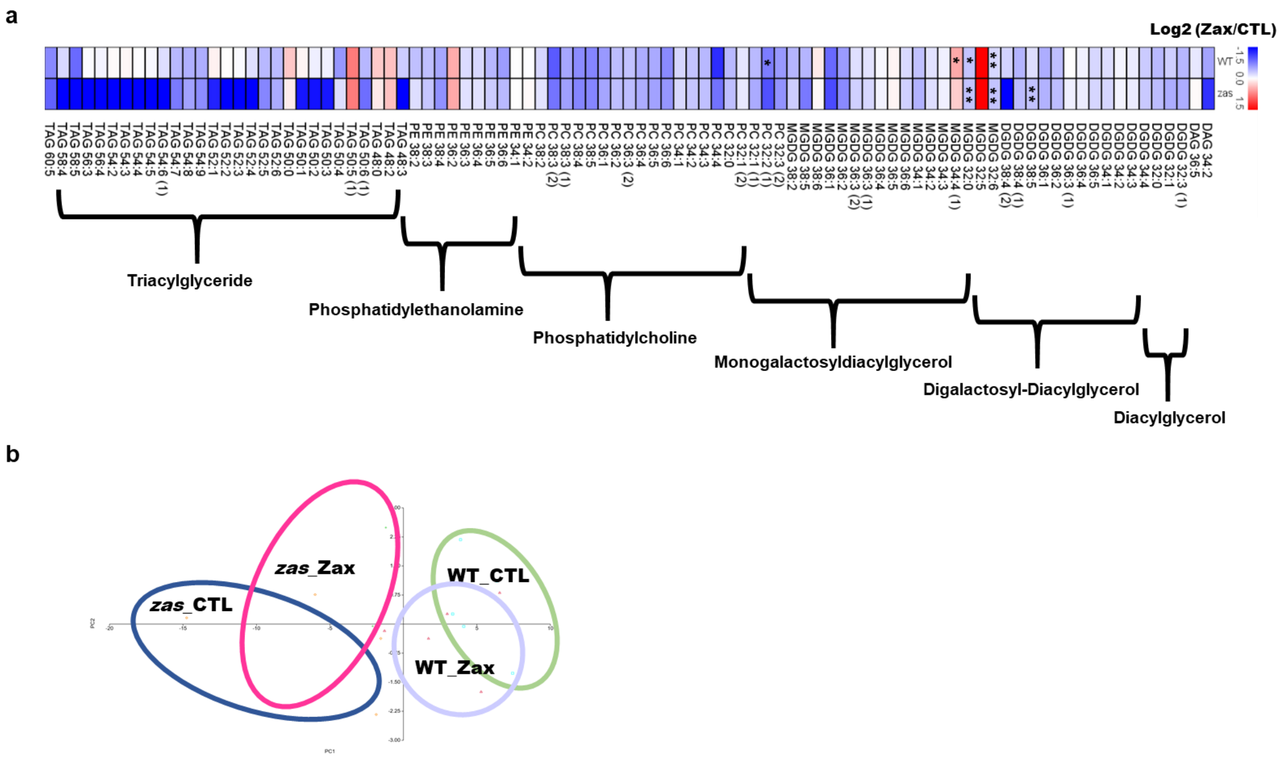

Supplementary Figure 13 The lipids profile in WT and *zas* root tissues upon 5 µM zaxinone treatment.

a Heat map of root tissues showing relative accumulation of each metabolite as compared to those in control plants. For each metabolite, the value of the corresponding wild type was set to 1, *n*≥4 biological replicates. Asterisks indicate statistically significant differences as compared to control by *t*-test (*P < 0.05, **P < 0.01). Color represented as Log_2 (FoldChange). **b** Principal component analysis (PCA) of root metabolites was performed using Past3 software. CTL, control; Zax, zaxinone.

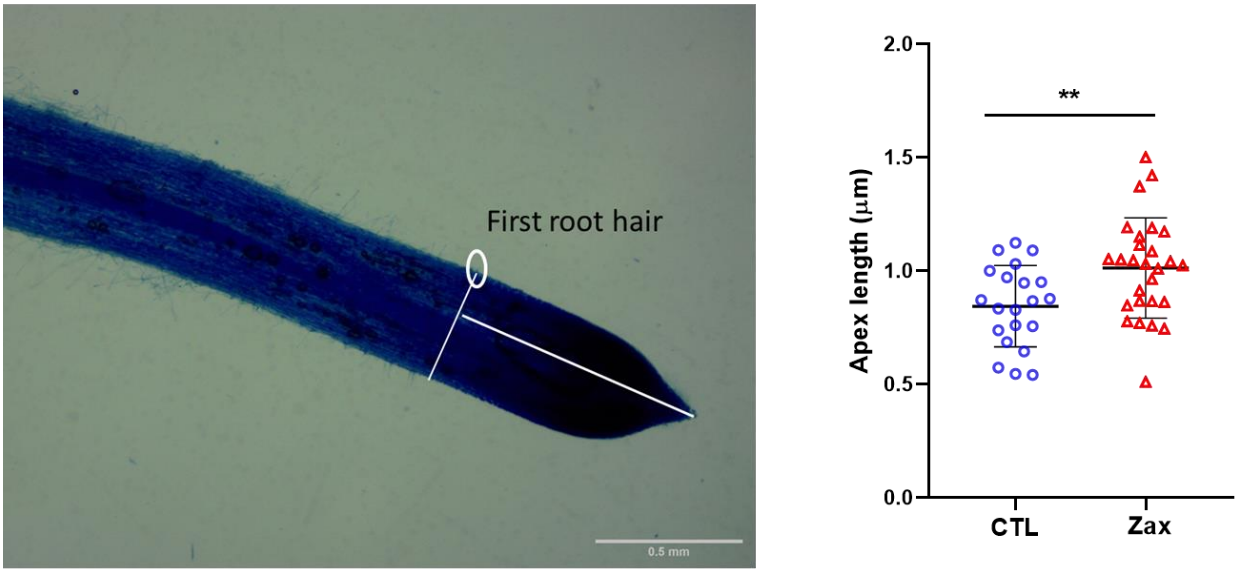

Supplementary Figure 14 Root apex analysis of WT rice plants.

Root apex length stained with 0.1% Cotton Blue, and the analysis was performed for the segment between the root tip and the first root hair (cell division zone + cell elongation zone). Bar presented as mean ± SD, $n \geq 20$ biological replicates. Asterisks indicate statistically significant differences as compared to control by *t*-test (**$p < 0.01$). CTL, control; Zax, zaxinone. Scale bar: 500 µm.

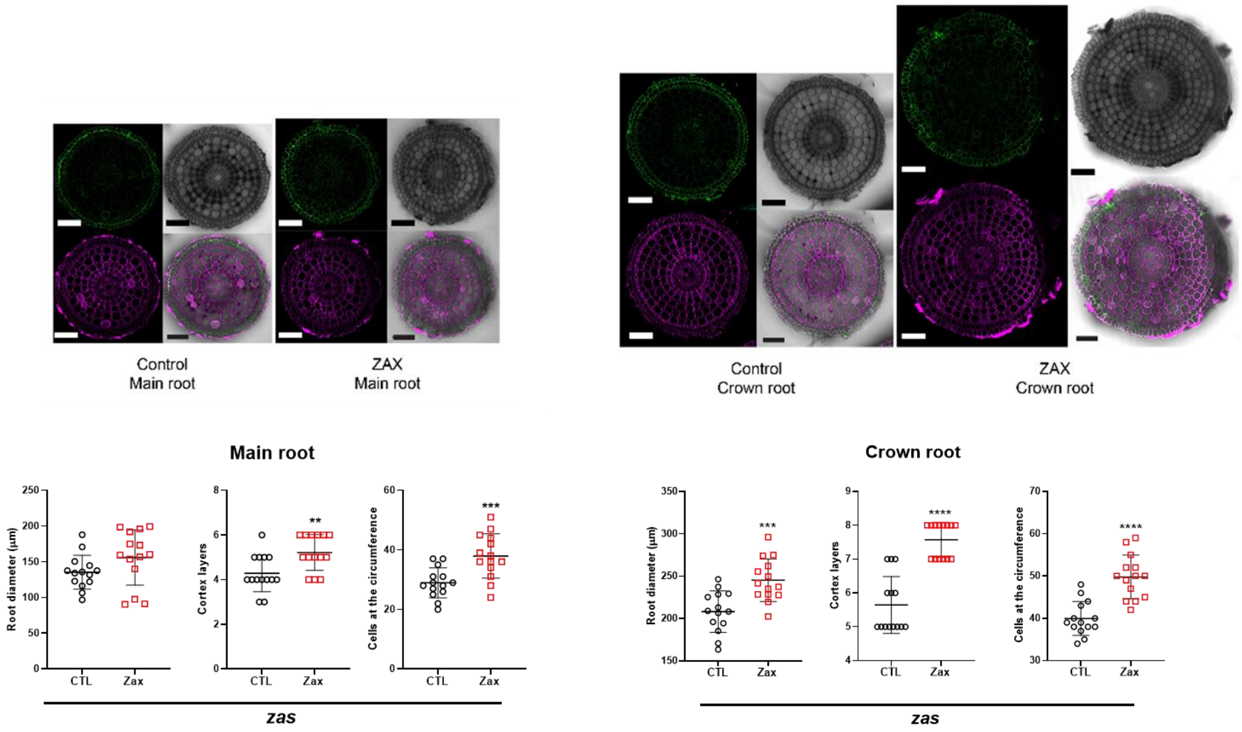

Supplementary Figure 15 Root cross section of *zas* mutant.

Cross-section of the mock and zaxinone treated roots stained with SCRI Renaissance 2200. Magenta indicates the cell wall staining; green shows the auto-fluorescence marking lignin and suberin deposition. Example of cell layer and circumference cell number count are indicated in the cross section **4d**. Bar presented as mean ± SD, n=14 biological replicates. Asterisks indicate statistically significant differences as compared to control by t-test (***$p < 0.001$; ****$p < 0.0001$). CTL, control; Zax, zaxinone. Scale bar: 50 µm.

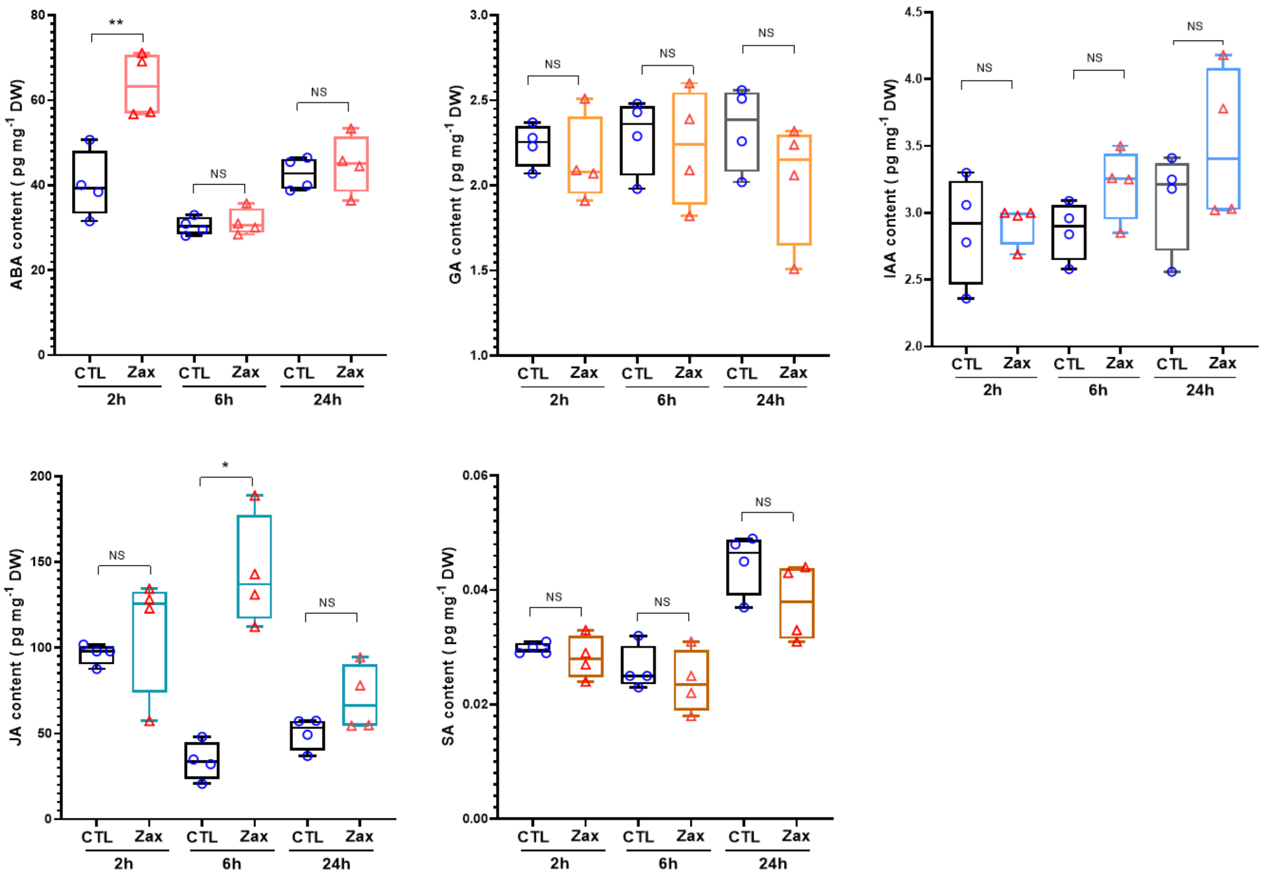

Supplementary Figure 16 Quantification of plant hormones (ABA, GA, IAA, JA, and SA) in WT root tissues upon zaxinone treatment.

Box plot presented as Min to Max, *n*=4 biological replicates. Asterisks indicate statistically significant differences as compared to control by *t*-test (*$p < 0.05$, **$p < 0.01$). CTL, control; Zax, zaxinone.

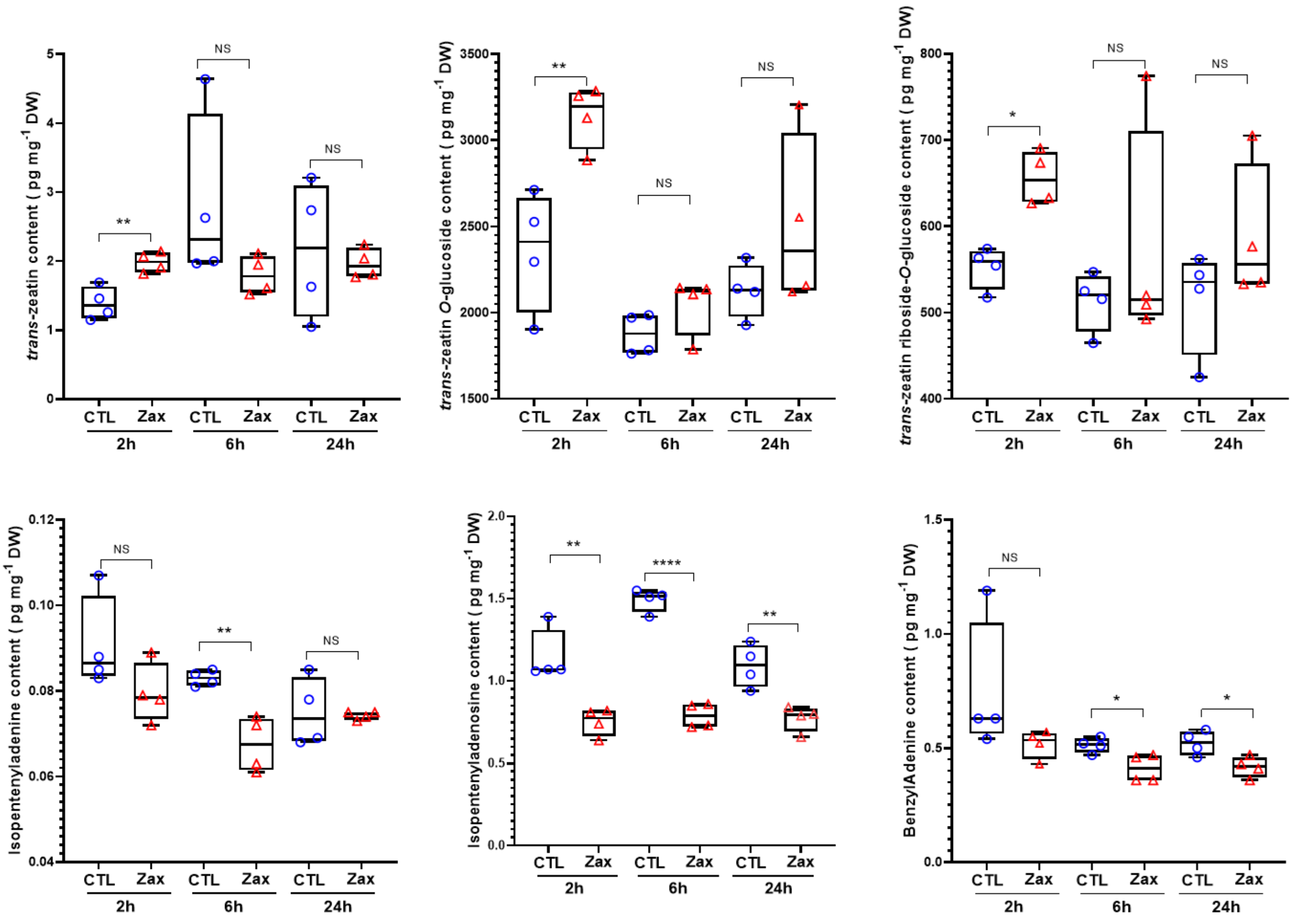

Supplementary Figure 17 Cytokinins (CKs) profiles in rice root tissues.

Quantification of cytokinins in a time course treatment of zaxinone in WT plants. Box plot presented as Min to Max, *n*=4 biological replicates. Asterisks indicate statistically significant differences as compared to control by t-test (**p* < 0.05, ***p* < 0.01; ****p* < 0.001; *****p* < 0.0001). CTL, control; Zax, zaxinone.

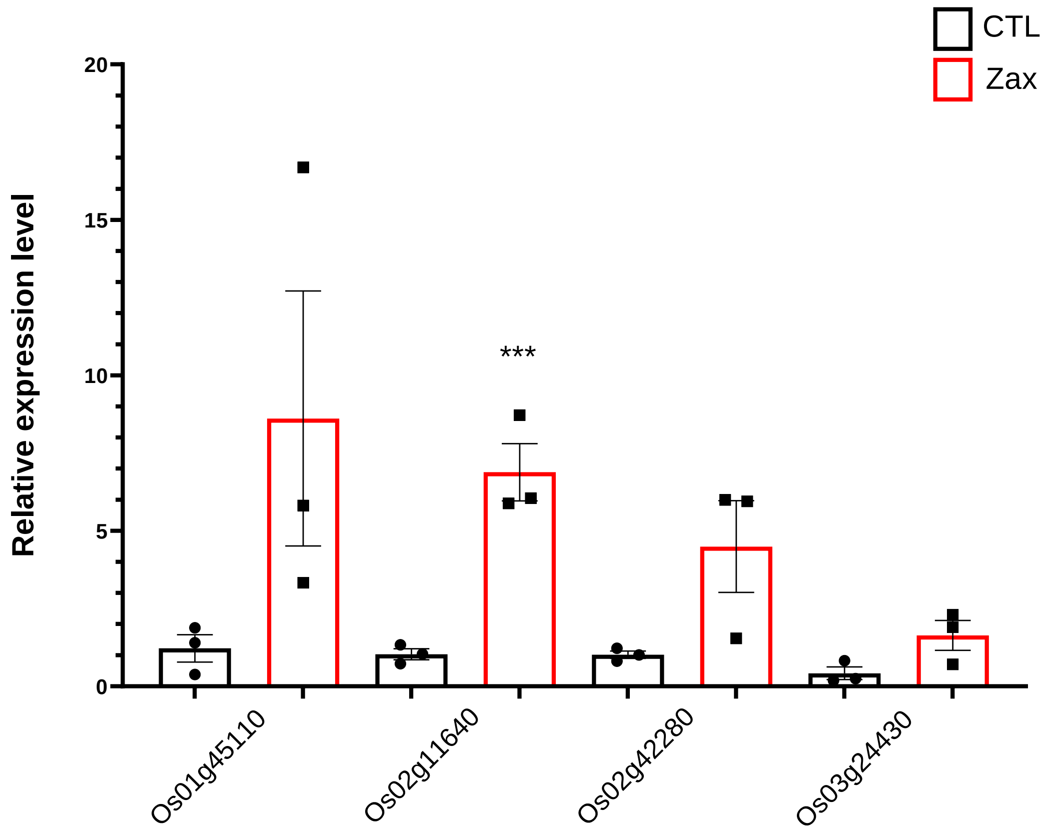

Supplementary Figure 18 Validation of cytokinin-glucosyltransferase genes by q-RTPCR in WT plant roots.

Four genes identified from the KEGG pathway, which are likely involved in cytokinin glycosylation in roots (Supplementary Data 5), and validated these using q-RT-PCR analysis of the same samples used for the RNAseq. Bar presented as mean ± SEM, *n*=3 biological replicates. Asterisks indicate statistically significant differences as compared to control by *t*-test (**p* < 0.05, (***p* < 0.01, ****p* < 0.001). CTL, control; Zax, zaxinone.

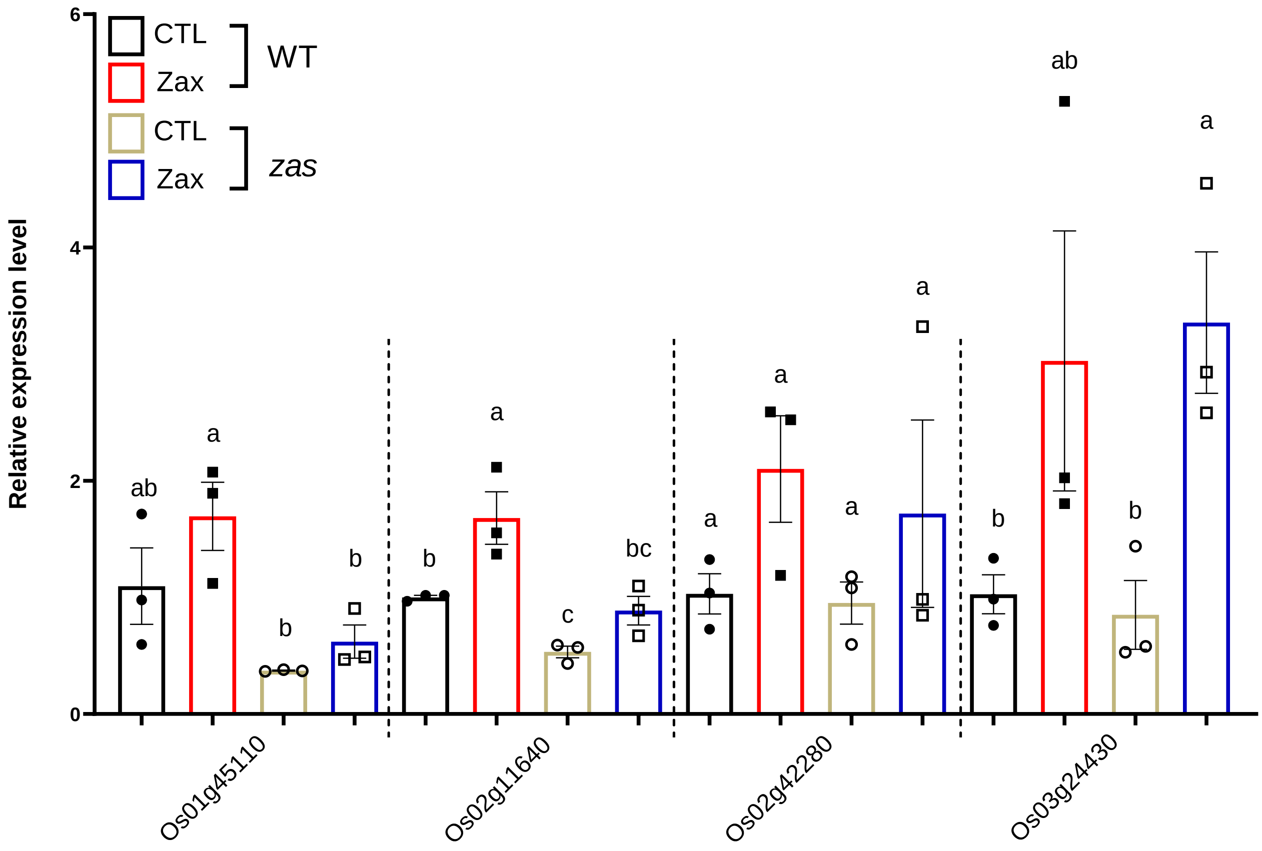

Supplementary Figure 19 Transcript analysis of root tissues upon 6 h zaxinone treatment in the roots of WT and *zas* mutant.

Four genes identified from the KEGG pathway, which are likely involved in cytokinin glycosylation roots (Supplementary Data 5). Bar presented as mean ± SEM, *n*=3 biological replicates. Statistical analysis was performed by one-way analysis of variance (ANOVA) and Tukey's post hoc test. Different letters denote significant differences ($p < 0.05$). CTL, control; Zax, zaxinone.

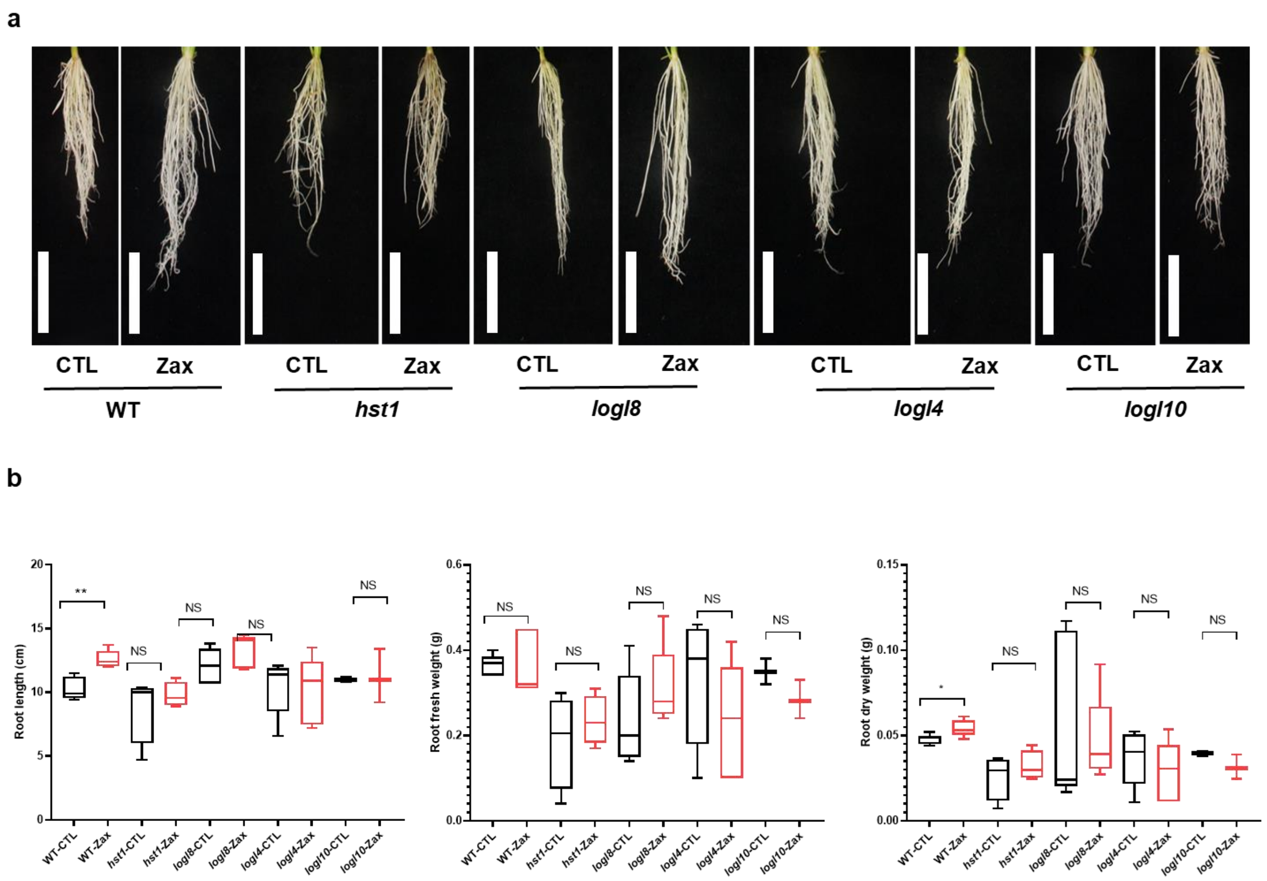

Supplementary Figure 20 Characterization of the effect of zaxinone on cytokinin biosynthesis and regulatory mutants

Effect of zaxinone (2.5 µM) on root phenotype of seedlings of TN67 wild-type and cytokinin biosynthetis/regulatory mutants. The seedlings were grown hydroponically. Asterisks indicate statistically significant differences as compared to control by t-test (*$p < 0.05$, **$p < 0.01$; NS, non-significant). Box plot presented Min to Max; n=5 biological replicates in WT, *logl4,* and *logl8*. n=4 biological replicates in hst1. $n \geq 2$ biological replicates in *logl10*. CTL, control; Zax, zaxinone. *hst1*, HITOMEBORE SALT TOLERANT 1 (also known as B-type response regulator designated OsRR22); *log1*, LONELY GUY-like. Scale bar: 5 cm.
