# Supplementary material for: Multi-omics approaches explain the growth-promoting effect of the apocarotenoid growth regulator zaxinone in rice
Source: Commun Biol. 2021 Oct 25;4:1222. doi: 10.1038/s42003-021-02740-8 (PMC8545949; doi:10.1038/s42003-021-02740-8)
Supplement: Supplementary file 2 — Description of Additional Supplementary Files [file 42003_2021_2740_MOESM2_ESM.pdf]

Description of Additional Supplementary Files

File name: Supplementary Data 1

Description: Differentially expressed genes (DEGs) analysis of root tissues in response to zaxinone.

File name: Supplementary Data 2

Description: Gene Ontology (GO) enrichment analysis of all the differentially expressed genes (DEGs). Gene sets were selected at FDR<0.05 and analyzed by Panther-Gene list analysis (http://pantherdb.org/). GO categories for each function were sorted by decreasing order of evidence, based on the GO enrichment test P-value.

File name: Supplementary Data 3

Description: Selected differentially expressed gene sets related to sugar metabolism at 6 hr. Metabolic genes and pathways were based on the KEGG analysis and the OyzaCyc 6.0 database available on the Plant Metabolic Network (PMN; https://pmn.plantcyc.org/organism-summary?object=ORYZA).

File name: Supplementary Data 4

Description: Differentially expressed genes (DEGs) analysis of shoot tissues in response to zaxinone.

File name: Supplementary Data 5

Description: Selected differentially expressed gene sets related to cytokinin-glucosyltransferase at 24 hr. Metabolic genes and pathways were based on the KEGG analysis and the OyzaCyc 6.0 database available on the Plant Metabolic Network (PMN; https://pmn.plantcyc.org/organism-summary?object=ORYZA).

File name: Supplementary Data 6

Description: Relative amounts of primary metabolites annotated by GC-MS analysis in WT rice root and shoot tissues.

File name: Supplementary Data 7

Description: Relative amounts of primary metabolites annotated by GC-MS analysis in WT, *zas*, and *d17* root tissues.

File name: Supplementary Data 8

Description: Primer sequences used in this study.

File name: Supplementary Data 9

Description: RNAseq sample list.

File name: Supplementary Data 10

Description: Source data of main figures.

File name: Supplementary Data 11

Description: Source data of supplementary figures.
